# (Trifluoromethoxy)Phenylboronic Acids: Structures, Properties, and Antibacterial Activity

**DOI:** 10.3390/molecules26072007

**Published:** 2021-04-01

**Authors:** Agnieszka Adamczyk-Woźniak, Jan T. Gozdalik, Ewa Kaczorowska, Krzysztof Durka, Dorota Wieczorek, Dorota Zarzeczańska, Andrzej Sporzyński

**Affiliations:** 1 Faculty of Chemistry, Warsaw University of Technology, Noakowskiego 3, 00-664 Warsaw, Poland; jgozdalik@ch.pw.edu.pl (J.T.G.); ewak@ch.pw.edu.pl (E.K.); kdurka@ch.pw.edu.pl (K.D.); 2 Faculty of Chemistry, University of Opole, Oleska 48, 45-052 Opole, Poland; dorwieczorek@uni.opole.pl; 3 Faculty of Chemistry, University of Gdańsk, Wita Stwosza 63, 80-308 Gdańsk, Poland; dorota.zarzeczanska@ug.edu.pl; 4 Faculty of Agriculture and Forestry, University of Warmia and Mazury, Oczapowskiego 8, 10-719 Olsztyn, Poland; andrzej.sporzynski@uwm.edu.pl

**Keywords:** boronic acids, trifluoromethoxy, OCF_3_, isomers, antibacterial, NMR, X-Ray, DFT

## Abstract

Three isomers of (trifluoromethoxy)phenylboronic acids were studied in the context of their physicochemical, structural, antimicrobial and spectroscopic properties. They were characterized by ^1^H, ^13^C, ^11^B and ^19^F NMR spectroscopy. The acidity of all the isomers was evaluated by both spectrophotometric and potentiometric titrations. The introduction of the -OCF_3_ group influences the acidity, depending, however, on the position of a substituent, with the *ortho* isomer being the least acidic. Molecular and crystal structures of *ortho* and *para* isomers were determined by the single crystal XRD method. Hydrogen bonded dimers are the basic structural motives of the investigated molecules in the solid state. In the case of the *ortho* isomer, intramolecular hydrogen bond with the -OCF_3_ group is additionally formed, weaker, however, than that in the analogous -OCH_3_ derivative, which has been determined by both X-Ray measurements as well as theoretical DFT calculations. Docking studies showed possible interactions of the investigated compounds with LeuRS of *Escherichia coli.* Finally, the antibacterial potency of studied boronic acids in vitro were evaluated against *Escherichia coli* and *Bacillus cereus.*

## 1. Introduction

Fluorinated arylboronic acids constitute an important group of compounds with a wide range of applications [1]. The introduction of a fluorine atom directly to the aromatic ring or to its substituents usually increases the Lewis acidity of these compounds, which is important in terms of their applications as well as stability [2]. The literature on arylboronic compounds with fluorine substituents is quite extensive and covers such areas as: acidity of the compounds [3,4], structural studies [5,6,7], NMR characterization [7,8], or equilibria in solutions [9,10,11] as well as antimicrobial activity [10,11]. The extreme electronegativity of the fluorine substituent induces a strong withdrawing inductive effect, whereas the resonance effect of its lone-pair electrons allows the fluorine atom to be considered as a π-electron donor as well [12]. In contrast, the perfluoroalkyl groups (e.g., -CF_3_) display only an electron-withdrawing effect, which should result in higher acidity of the corresponding phenylboronic acid in comparison with their fluorine substituted analogues. It is not, however, the case of the *ortho* isomer, primarily due to the steric hindrance. Very little is known, however, about the properties of boronic acids containing the -OCF_3_ group. The syntheses of all isomers of (trifluoromethoxy)phenylboronic acids have been previously reported [13,14,15]. The title compounds have been numerously used in synthetic applications, mostly enabling the introduction of the trifluoromethoxyphenyl fragment [16,17,18,19,20,21,22,23,24,25,26,27,28].

**Figure 1:**
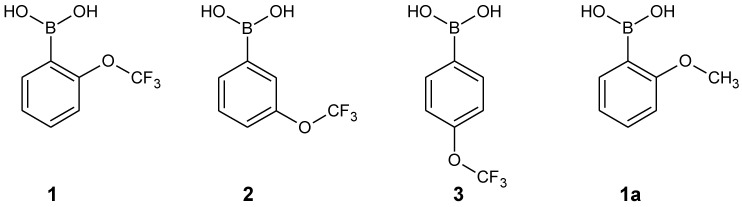
The compounds investigated in the present work.

In addition, Hansen and coworkers investigated the selectivity of d-glucose binding by *ortho* substituted phenylboronic acids. They found that for the *ortho*-(trifluoromethoxy)phenylboronic acid (**1**), d-glucose is bound more strongly than d-fructose, which is quite uncommon for phenylboronic acids and can be useful in the diagnostics of diabetes mellitus [29]. Moreover, Golovanov et al. described the use of triolborane with the *para*-trifluoromethoxy group as a scavenger for removing an excess of boronic acid in the Suzuki–Miyaura coupling [30]. Despite those numerous applications, no systematic investigation of the properties of trifluoromethoxy phenylboronic acids have been reported so far. The aim of the present work is to fully characterize phenylboronic acids with the -OCF_3_ substituent (Figure 1) both in solution and in the solid state as well as to assess their acidity and potential as antibacterial agents. The properties of **1**–**3** have been compared with their analogues, including compound **1a**.

## 2. Results and Discussion

### 2.1. Acidity and Stability

Acidity constants of all the isomers (**1**–**3**) were determined by both potentiometric and spectrophotometric methods and compared with values previously reported for other monosubstituted phenylboronic compounds (Table 1). The values obtained by both methods are consistent.

**Table 1:** p*K*_a_ values of the compounds **1**–**3** (X = OCF_3_) in comparison with other mono-substituted phenylboronic acids (X = OCH_3_, CF_3_ and F). Values determined potentiometrically are given in italics.

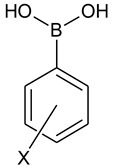	**X**	***ortho***	***meta***	***para***	**Ref.**
	OCF_3_	9.51 ± 0.04 *9.49 ± 0.08*	7.79 ± 0.02 *7.96 ± 0.07*	8.11 ± 0.04 *8.03 ± 0.07*	this work
	OCH_3_	*9.31 ± 0.02* [31]	*8.46* [32]	No data	[31,32]
	CF_3_	9.45 ± 0.01 *9.58 ± 0.16*	7.88 ± 0.01 *7.85 ± 0.05*	7.82 ± 0.01 *7.90 ± 0.10*	[7]
	F	7.89 ± 0.01 *7.85 ± 0.07*	8.09 ± 0.01 *8.15 ± 0.11*	8.77 ± 0.01 *8.71 ± 0.10*	[3]

The influence of the introduction of the trifluoromethoxy substituent on p*K*_a_ depends on its position in the phenyl ring. According to expectations, the acidity of *meta* (**2**) and *para* (**3**) isomers is higher than that for the unsubstituted phenylboronic acid: their p*K*_a_ values are ca. one unit lower than that of a parent PhB(OH)_2_, which is 8.8 [3]. This is due to the inductive effect of the -OCF_3_ group, which in comparison with the trifluoromethyl substituent (-CF_3_ group) is slightly diminished by the resonance effect of an oxygen atom. Needless to say, that phenylboronic acids are Lewis, not Broensted acids. Therefore, despite the strong inductive effect of the -OCF_3_ group, the acidity of the *ortho* isomer (**1**) is much weaker than that for the unsubstituted phenylboronic acid. This can be explained by the influence of a bulky substituent proximal to the boronic group, which decreases the acid strength due to the steric inhibition of the formation of the tetrahedral boronate ion. A similar effect was observed for *ortho*-trifluoromethylphenylboronic acid [7]. It is worth underlining that the opposite effect occurs in case of *ortho*-F acid, for which increased acidity is caused by the formation of an intramolecular B-O-H^...^F hydrogen bond [5,33]. The comparison of the values of the series of investigated compounds with their CF_3_ substituted analogues shows that the resonance effect of the oxygen atom is small—it occurs visibly only for the *para* isomer (**3**). For the remaining isomers, the values of both types of compounds are practically identical. When comparing the acidity of **1** with its -OCH_3_ analog (**1a**), it can be concluded that the steric repulsion predominates inductive effect, since the acidity of **1** (p*K*_a_~9.5) is even slightly lower than that of **1a** (p*K*_a_~9.3) which contains an electrodonating substituent. It is worth noting that the literature p*K*_a_ value of **1** is considerably lower and equals to 8.3 [29]. Since Hansen and co-workers dissolved **1** in 0.025 M NaOH and titrated such a solution with HCl, it was assumed that the observed discrepancy could result from the possible instability of **1** in basic solution [3]. To rule this out, the stability of **1**–**3** in NaOH solution was investigated by UV–Vis spectroscopy (see Supplementary Materials for spectra) showing no changes in the spectra recorded after 2 h at room temperature for **1** and after 1.5 h for **2** and **3**. Reproducing directly the literature protocol [29] gave the p*K*_a_ = 9.46 ± 0.08, which is consistent with those reported in Table 1.

### 2.2. NMR Characterization

**Figure 2:**
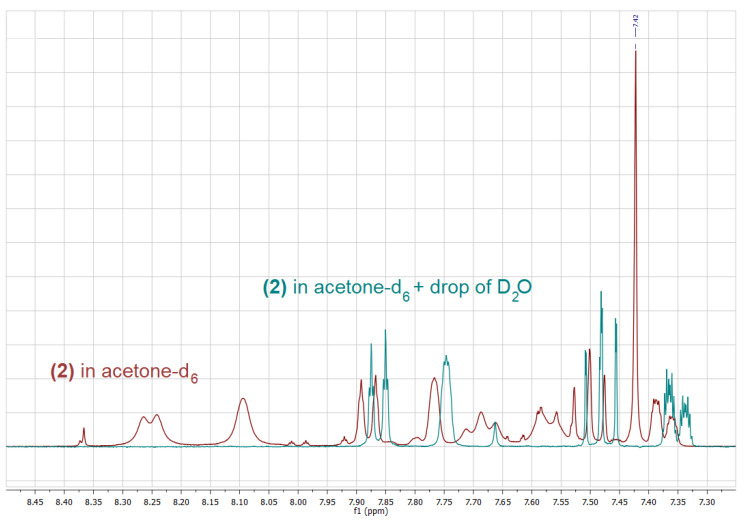
^1^H-NMR spectrum of the on-shelf sample of **2** in acetone-d_6_ (red) and after the addition of a drop of D_2_O (blue).

A number of one- and two-dimensional experiments were conducted to fully characterize all isomers of phenylboronic acids substituted with the OCF_3_ group (**1**–**3**) in solution. Acetone-d_6_ was chosen as a solvent for NMR measurements due to a very good solubility of all the title compounds in this solvent, which was especially important for carbon experiments, requiring a sufficient concentration of the sample. For all the isomers, ^1^H, ^11^B, ^13^C, ^19^F NMR and ^1^H-^13^C HSQC spectra were recorded. Additionally, for the *ortho* isomer (**1**) a ^1^H-^1^H COSY experiment was performed, enabling the unambiguous assignment of all the signals in the ^1^H NMR spectrum. It is worth noting that the on-shelf sample of **2** contained big amounts of the corresponding boroxine, which was removed by the addition of a drop of D_2_O to the sample, resulting in a clear phenylboronic acid spectrum (Figure 2).

**Figure 3:**
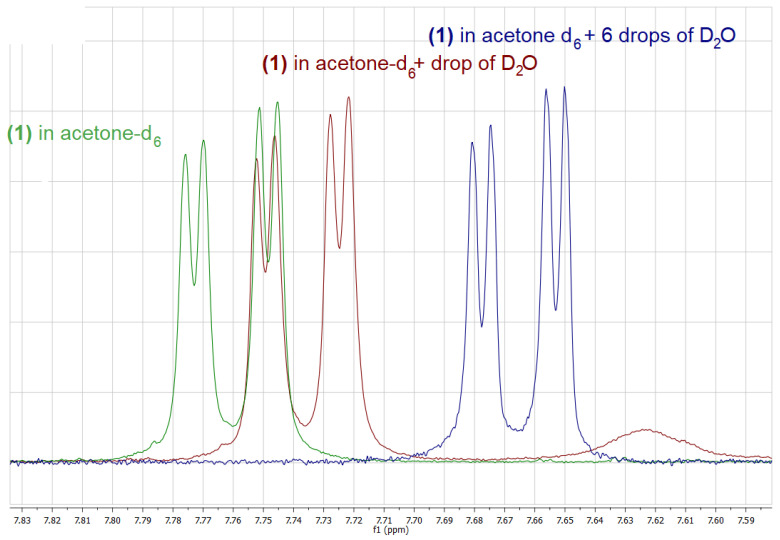
Fragment of the ^1^H-NMR spectra of **1** in acetone-d_6_ (green), after the addition of a drop of D_2_O (**red**) and after the addition of 6 drops of D_2_O (blue).

On the contrary, in the case of **1** and **3**, only boronic acids signals, including singlets corresponding to the B(OH)_2_ group have been observed in spectra recorded in (CD_3_)_2_CO solution. Interestingly, the addition of a drop of D_2_O, results not only in the hydrolysis of the boroxine and the disappearance of its signals as in the case of **2**, but also in a considerable shift of the signals corresponding to the boronic acid itself, as it was observed for both **2** and **1** (Figure 2 and Figure 3).

Upon the addition of a small amount of D_2_O to the boronic acid’s solution in deuterated acetone, two further changes can be observed: (i) the signal corresponding to the B(OH)_2_ decreases its intensity due to a partial exchange of the OH groups with OD groups, (ii) the respective signal changes its chemical shift considerably, which results from the change in the structure as well as the concentration of hydrogen bonded species. Upon the addition of an appropriate amount of D_2_O, the above-mentioned signal disappears. Interestingly, the addition of D_2_O influences also chemical shifts of signals corresponding to aromatic protons; however, this effect is much smaller in that case (Figure 2 and Figure 3).

**Figure 4:**
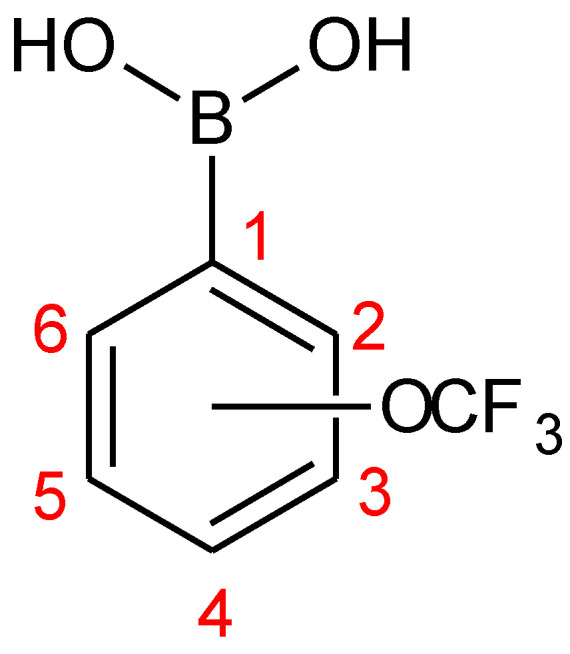
Numbering of atoms in the aromatic ring of investigated boronic acids (**1**–**3**).

Another interesting feature of the ^1^H-NMR spectra of all the isomers are long-range couplings of aromatic protons resulting in complex multiplets. These couplings have been confirmed for **1** by means of a ^1^H-^1^H COSY experiment. In addition, the fluorine atoms from the trifluoromethoxy group couple with aromatic ring protons, splitting signals corresponding to neighboring protons, i.e., H3 in the case of **1**, H2 and H4 and in the case of **2** as well as H3 and H5 in the case of **3**, respectively (see Figure 4 for atoms numbering). Table 2 shows the ^1^H chemical shifts observed in the spectra of **1**–**3** in acetone-d_6._

The most characteristic signal in the ^13^C-NMR spectra of all the investigated compounds are quartets corresponding to the OCF_3_ group with a big coupling constant (*^1^J_C-F_*) of about 255 Hz. The signal of the carbon atom neighboring the OCF_3_ is also coupled with fluorine atoms, however the observed coupling constant (*^3^J_C-F_*) is much smaller and equals to 1.7 Hz in all cases (**1**–**3**). Only in the case of **1**, a longer-range coupling (*^4^J_C-F_*) is manifested with a quartet with a coupling constant of 1.0 Hz. All the observed C-F coupling constants are in agreement with the values recorded for α,α,α-trifluoroanisole, which spectra in acetone-d_6_ were measured for comparison (see Table 3 for a list of ^13^C-NMR signals). In the HSQC spectra, strong correlation signals (four for **1**–**2** and two for **3**) were detected, enabling the pairing of the signals in ^1^H and ^13^C-NMR resulting from neighboring H and C atoms. The observed ^11^B-NMR chemical shifts (Table 4) are typical for tri-coordinated boronic species and analogous for all the isomers (**1**–**3**) showing lack of interactions between the boronic group and the trifluoromethoxy group. Interestingly, in the ^19^F NMR spectra a long-range coupling with aromatic protons is observed, resulting in a doublet for **1**, pseudo triplet (doublet of doublets) for **2** and a triplet for **3**. The analogous long-range couplings with fluorine nucleus are observed in corresponding signals in proton spectra.

### 2.3. Molecular Structure and Conformation

**Figure 5:**
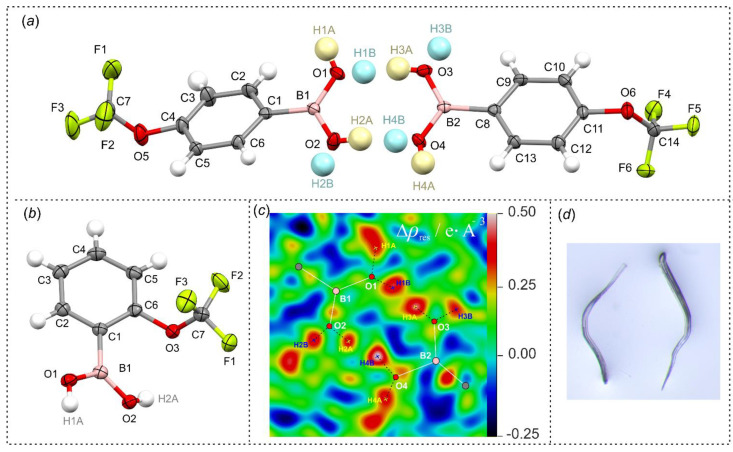
(**a**,**b**) Molecular structures of **3** and **1**. Ellipsoids are drawn on a 50% probability level. Disordered hydrogen atoms (**a**) are presented with two different colors. (**c**) Residual density map for the hydrogen-bonded dimer in **3** reconstructed without hydrogen atoms on B(OH)_2_ groups. (**d**) Photo of single crystals of **1**; plastic bending was observed under small force.

Crystal structures of *ortho*- and *para*-(trifluoromethoxy)phenylboronic acids (**1** and **3**) were analyzed using the single-crystal X-ray diffraction method. Views of the molecular structures together with atom labelling scheme are shown in Figure 5. Despite numerous attempts, we were unable to obtain single crystals of isomer **2**. The position of trifluoromethoxy substituent only slightly affects the C–B and O–B distances (Table 5). However, as indicated by C2–C1–B1–O1 dihedral angle (*τ*_C2C1B1O1_), studied molecules are characterized by different twist angle of boronic groups along C–B bond. In the case of **3-A** and **3-B** boronic groups deviate by 16.9(1)° and 23.4(1)° from aromatic ring plane, respectively. Such a twist is characteristic for a number of arylboronic acids as it allows one to avoid sterical repulsion between aromatic moieties from molecules arranged in side hydrogen-bond interactions [5]. In turn, the conformation of molecule **1** carrying the OCF_3_ substituent in the *ortho* position is predefined by the O2–H2A…O3 intramolecular hydrogen bond (*d*_O…O_ = 2.839(2) Å; *d*_H…O_ = 2.38(2) Å) [34]. Thus *τ*_C2C1B1O1_ dihedral angle is higher and equals to 26.5(1)°. The appearance of an intramolecular hydrogen bond also stabilizes the *syn*-*anti* conformation of boron-bonded hydroxyl groups. A different situation is observed for **3**, where boronic hydrogen atoms are disordered and can occupy two different sites, as it can be clearly visible on a Fourier residual electron density map (Figure 5c) [35].

**Figure 6:**
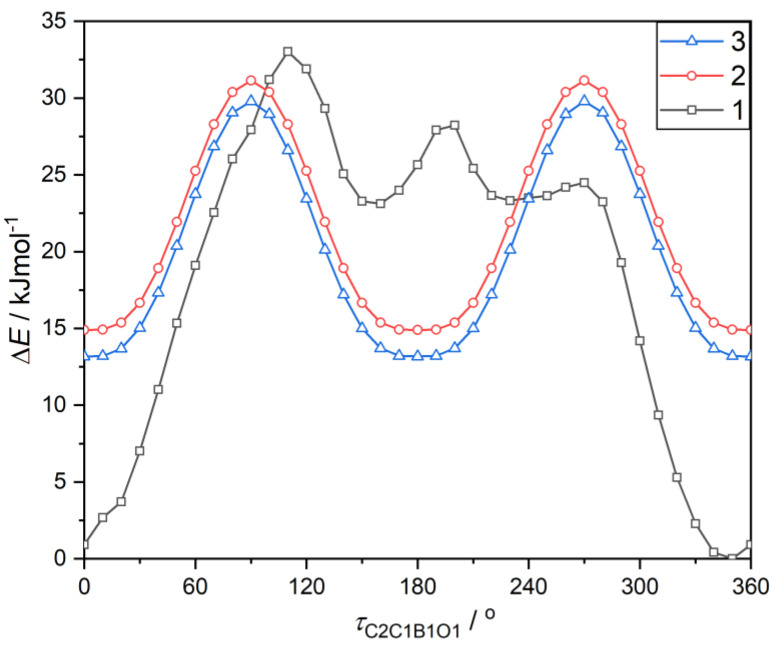
The energy profile for the rotation of boronic groups in respect to the phenylene ring fragment. The energy of all compounds were referred to the most stable conformation of the *ortho* isomer (Δ*E*(*τ*_C2C1B1O1_ = 350°) = 0 kJ mol^−1^). The energy rotation profile for the *ortho* isomer is asymmetric, which results from the twist of the OCF_3_ group around the O–C bond (*τ*_C6C1O2C7_ = 65.6°), leading to the asymmetrical surrounding of boronate *syn* and *anti* conformed OH groups.

Theoretical calculations performed at M062X [36]/6–311 + G(d,p) [37] level of theory revealed that the rotation barrier (*E*_rot_) for the twist of boronic group is 16.2 kJ mol^−1^ and 16.6 kJ mol^−1^ for *para* (**3**) and *meta* (**2**) O-CF_3_ substituted phenylboronic acids, respectively (Figure 6). This resembles previously reported values for arylboronic compounds [35]. Due to the presence of an intramolecular hydrogen bond, the energy barrier is much higher for the *ortho* isomer (*E*_rot_ = 33.0 kJ mol^−1^). Concordantly, molecule **1** is more stable than **2** and **3**. Taking into account the boronic group resonance stabilization effect, the intramolecular hydrogen bond energy was estimated to 17 kJ mol^−1^. This is in general agreement with the value calculated from topological features of electron density at the bond critical point (BCP) using the Espinosa–Lecomte estimation (*E*^EL^_int_ = 19.0 kJ mol^−1^, Table 6) [38]. Interesting conclusions can be derived from the comparison with 2-methoxyphenylboronic acid (**1a**) [39]. As indicated by theoretical calculations, hydrogen bond interaction with the methoxy group in **1a** is more intense (*E*^EL^_int_ = 29.3 kJ mol^−1^). This is accompanied by the shortening of the donor–acceptor O…O distance (*d*_O…A_ = 2.817 Å in **1**; *d*_O…A_ = 2.719 Å in **1a**) and an increased amount of electron density at BCPs ($\rho \left(r_{\mathrm{BCP}}\right)$ = 0.136 e Å^3^ in **1**; $\rho \left(r_{\mathrm{BCP}}\right)$ = 0.177 e Å^3^ in **1a**), (Table 6). The observed differences are the result of strong inductive effect of fluorine atoms and polarization of C–F bonds, which decrease oxygen Lewis basicity. The second order perturbation theory analysis within the natural bond orbital (NBO) [40] method clearly shows that the interaction between oxygen-centered donor lone pairs, LP1 and LP2 orbitals, associated with lone pairs and antibonding Lewis-acceptor BD*(C–F) orbitals in **1** is energetically higher than respective interactions with BD*(C–H) antibonding orbitals in **1a** (Table 7). This is partially counterbalanced by a slightly decreased OCF_3_ resonance stabilization effect with the aromatic ring, which is manifested by a lower interaction energy of LP1 with antibonding C-C π orbital. Concordantly with this observation, the aromaticity of **1**, quantified by the nucleus-independent chemical shifts (NICS(1)) parameter [41], is higher with respect to **1a** (NICS(1) = −10.61 for **1**; NICS(1) = −10.38 for **1a**).

The analysis of the negative Laplacian function (*L*(**r**) = –∇^2^*ρ*(**r**)) around the OCH_3_ and OCF_3_ oxygen atoms provides two sets of (3, −3) critical points (CPs) corresponding to a local maximum of electron density (CC), which can be associated with the oxygen lone pairs. Interestingly, despite the fluorine inductive effect, the depletion of electron density around oxygen atom in **1** is less pronounced with respect to **1a** (Table 8). This can be rationalized by the presence of a stronger intramolecular hydrogen bond in the latter system and electron transfer to hydrogen atoms from B(OH) group. Along with this observation, the NBO analysis shows that the occupancy of oxygen LP1 orbital arranged in HB formation decreases from 1.900 e in **1** to 1.868 e in **1a**, while the occupancy of BD*(O–H) antibonding orbital increases from 0.011 e to 0.016 e. On the contrary, the occupancy of LP2 Lewis donor orbital, which mostly is arranged in the C–F or C–H antibonding orbitals overlap, is lower in **1** than in **1a**.

### 2.4. Supramolecular Structure

**Figure 7:**
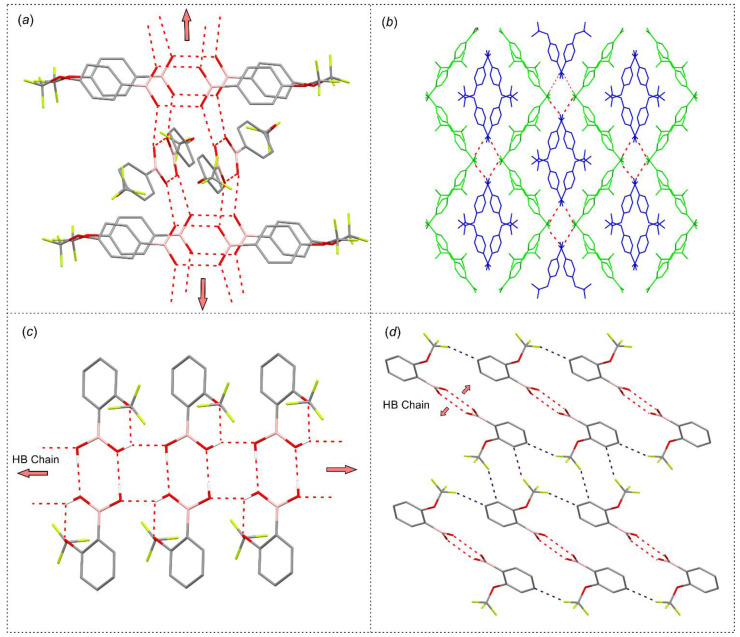
Packing diagrams showing the formation of main crystal motifs in structures (**a**,**b**) **3** and (**c**,**d**) **1**. O–H…O hydrogen bonds are depicted with red dashed lines, while C–H…F with deep blue dashed lines. (**b**) Two different colors are used to depict two symmetrically non-equivalent molecules **3-A** (blue) and **3-B** (yellow).

Compound **3** crystallizes in the *P*-1 triclinic space group of symmetry with two non-equivalent molecules in the asymmetric part of the unit cell denoted as **3-A** and **3-B**. According to expectations, they form a hydrogen-bonded dimer as it is typically encountered in a number of arylboronic acid structures. Likewise, the formation of such a hydrogen-bonded dimer is observed in structure **1**. The theoretical calculations performed at M062X/6-311 + G(d,p) level of theory revealed that the binding energy in both dimers equals to about 45 kJ mol^−1^. Such dimers are further connected by side hydrogen bonds, leading to the formation of two essentially different molecular chains. In the case of **1,** neighbored dimers are associated by two sets of symmetric hydrogen bonds arising from *anti*-conformed hydroxyl groups with the calculated interaction energy of 28.4 kJ mol^−1^ in each dimer. The propagation of this supramolecular synthon along [100] direction leads to the formation of a characteristic molecular ladder (Figure 7c) [43]. In turn, in crystal structure of **3** hydrogen bonded dimers are grouped in pairs and further connected by side hydrogen bonds to form a characteristic supramolecular chain, where subsequent dimeric pairs are rotated by 58.3° and propagate along [001] crystal direction in an alternate fashion (Figure 7a). The estimated energy of such a dimer is 25.5 kJ mol^−1^. This structural motif resembles the hydrogen bonded chain found in the crystal structure of unsubstituted phenylboronic acid [44]. The higher levels of supramolecular assembly in both structures are dominated by the interactions with fluorine atoms from the -CF_3_ group. In particular, in the case of **3**, the molecules from neighbored chains are associated by weak C–H…F and F…B interactions. Similarly, the aggregation of molecular chains in **1** results mainly from weak C–H…F interactions reinforced by C_ar_(π)…B interactions. Finally, an interesting observation can be made while analyzing the mechanical features of single crystals. Compound **3** forms typical cuboidal crystals, which are easily crushed under applied force. In contrast, **1** crystallizes in the form of very tiny needles, which easily undergo mechanical plastic deformation under force (Figure 5d) [45].

### 2.5. Biological Activity

#### Docking Studies

**Figure 8:**
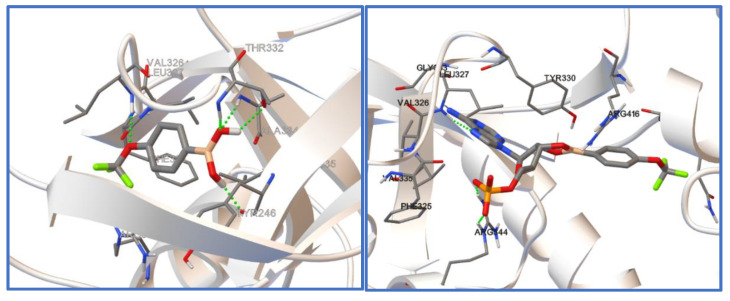
Compound **3** (**left**) and its AMP ester (**3-AMP**, **right**) docked into the binding pocket of *Escherichia coli* LeuRS.

It was recently shown by docking studies that the 5-trifluoromethyl-2-formylphenylboronic acid can bind to the active pocket of *Escherichia coli* LeuRS, resulting in its antibacterial activity determined in vitro [10]. Therefore, docking studies have been carried out to check the possibility of such a binding in the case of the investigated compounds (**1**–**3**). The *E. coli* LeuRS (4ARI) complex with a boronic ligand (AN2679) was taken from the protein databank [46] and used in docking studies. The initial boronic ligand was removed from the complex and the docking procedure exclusively covered the binding domain of the enzyme. To judge the binding of the investigated ligands (**1**–**3**), the binding energy was determined as a difference between the energy of a protein–ligand complex and the sum of the energy of a ligand and a protein. Table 9 contains the chosen results of docking studies. The binding energies show that both the ligands themselves as well as their esters with AMP dock into the binding pocket, proving possible interactions with the enzyme and potential activity. It is worth noting, however, that the resulting inhibition constants differ significantly (Table 9). Since the determined inhibition constants are much lower for the AMP esters than for the boronic molecules, the formation of the AMP ester seems to be crucial for a tight interaction with the enzyme and resulting activity. It needs to be underlined that all the determined inhibition constants are in the micromolar range, whereas in the case of previously studied boronic ligand, they were in the nanomolar range. Figure 8 shows compound **3** docked into the binding pocket of *Escherichia coli* LeuRS.

The antimicrobial activity of **1**–**3** in vitro was established using both the agar diffusion method as well as by determination of the minimal inhibitory concentration (MIC) values. DMSO was used as a negative probe, while streptomycin as a positive control. Gram-negative—*Escherichia coli* and Gram-positive—*Bacillus cereus* bacterial strains were chosen as the representatives of two microbiota of various susceptibility towards the action of antibiotics. The results are shown in Table 10 and Table 11, respectively. Interestingly, compound **1** revealed no influence on the growth of any of the investigated bacteria at any of the amounts applied. In the case of compounds **2** and **3**, a rather low if any activity against both bacterial strains was detected. However, similarly as in the case of the previously reported 5-trifluoromethyl-2-formylphenylboronic acid [10], *B. cereus* is a bit more vulnerable to **2** and **3** than *Escherichia coli*, with MIC values equal to 125 [μg/mL] (Table 11) and 250 [μg/mL] (Table 10), respectively.

## 3. Experimental

### 3.1. Materials

Compounds **1**–**3** were purchased from Flurochem. Purity: 98%.

### 3.2. pK_a_ Determination

The acid dissociation constants of the tested compounds were determined using two methods: potentiometric titration and spectrophotometric titration. The titrant in both experiments was sodium hydroxide solution prepared by dissolving solid sodium hydroxide in water. The concentration of the prepared NaOH solution was determined by potentiometric titration with a standard 0.1 M hydrochloric acid solution and was 0.0409 M. Potentiometric titrations were performed with a microtitration automatic Cerko-Lab system equipped with a Schott Blue line N16 pH electrode and a 1.0 mL Hamilton syringe. The pH glass-electrode was calibrated with four buffer solutions. The resolution of the voltage measurements was <0.1 mV. The compounds were dissolved in an aqueous solution with a constant ionic strength (0.1 M KCl). The concentrations of the boronic acid solutions were in the range of 2 × 10^−3^–8 × 10^−4^ M. The potential was recorded every 30 s. Spectrophotometric titrations were performed on a Perkin Elmer UV–Vis spectrophotometer Lambda 650, using quartz cuvettes of a 1 cm light pathlength. Spectrophotometric titration was performed under conditions analogous to those used for the potentiometric measurements. Spectral changes presented as a dependence *A* = f (pH) take into account a correction to the dilution of the analyte during the measurement. All measurements were performed at 298 K. The dissociation constants values obtained with the potentiometric method were calculated using a STOICHIO version CV EQUID computer program which uses the non–linear least-squares Gauss–Newton–Marquardt method for the fitting procedure [47,48,49].

The values obtained with the spectrophotometric method were calculated with the Henderson–Hasselbach equation implemented into Origin Lab software. It is based on the change in absorptions’ intensity as a function of pH of the solution [50].

### 3.3. Stability Studies

Solutions of **1** (0.00287 g), **2** (0.00279 g) and **3** (0.00305 g) in 10 mL of 0.025 M NaOH each were prepared at room temperature. The UV–Vis spectra was taken immediately after preparation and every 10 min for 2 h (compound **1**) and 1.5 h (compounds **2** and **3**).

### 3.4. NMR Spectroscopy

All NMR experiments (^1^H, ^11^B, ^13^C, ^19^F-NMR, ^1^H-^1^H COSY and ^1^H-^13^C HSQC), were performed on a Bruker Avance 300 MHz spectrometer. For proton and carbon spectra, solvents’ residual signals were used as internal references. For the ^11^B and the ^19^F spectra, BF_3_·Et_2_O and CCl_3_F were used as external references, respectively.

### 3.5. Crystal Structure Determination

The single crystals of **1** and **3** were prepared by crystallization from acetone and toluene solutions, respectively. To remove concomitant boroxines, a drop of water was added to each crystallization vial. They were left under perforated Parafilm^®^ to slowly evaporate the solvent. After 7–10 days the solvent evaporated completely, yielding single crystals of **1** and **3** suitable for X-ray diffraction studies. Despite numerous attempts, we did not manage to obtain single crystals of **2** of an appropriate size and quality. X-ray diffraction data sets for single crystals **1** and **3** were collected at 100 K on a SuperNova diffractometer equipped with an Atlas detector (Mo-*K*_α_ radiation, *λ* = 0.7107 Å). The data collection strategy was optimized using CrysAlisPro software [51]. Data reduction, analysis and integration was carried out using CrysAlisPro software. Absorption correction from crystal shape was applied. Structures were solved by direct methods using SHELXS-97 [42] and refined using SHELXL-2014 [52]. All non-hydrogen atoms were refined anisotropically. All C–H hydrogen atoms were placed in calculated positions with C–H distances of 0.95 Å and *U*_iso_(H) = 1.2*U*e_q_(C). In the case of OH hydrogen atoms, their position was established from difference density maps. Then, O–H distances were restrained to 0.84 Å, while the directionality of OH bonds was refined freely. The U_iso_ parameter was set to 1.5*U*_eq_ of oxygen atom. Crystallographic information files (CIFs) have been deposited with the Cambridge Crystallographic Data Centre as supplementary publications no. 2,044,318 (**1**), 2,044,319 (**3**). Selected crystal data collection, reduction and refinement are gathered below.

**1**: C_7_H_7_BF_3_O_3_, *M*_r_ = 205.93 a.u.; *T* = 100 K; monoclinic, *C*2/*c*, *a* = 21.7886(10) Å, *b* = 17.7996(4) Å, *c* = 9.7820(3) Å, *β* = 109.780(4)°; *V* = 3569.9(2) Å^3^; *d*_calc_ = 1.533 gcm^−3^; *μ* = 1.381 mm^−1^; F(000) = 1664; *θ* full: 67.7°; number of collected/independent/unique reflection (*R*_int_ = 1.76%) = 12,964/3696/3554; no. of parameters/restrains = 278/8; GOF = 1.272; R[F]/wR[F] (I ≥ 3σ(I)) = 5.97%/12.65%; Δ*ρ*^max/min^ = +0.34/−0.26 eÅ^3^.

**3**: C_7_H_7_BF_3_O_3_, *M*_r_ = 205.93 a.u.; *T* = 100 K; triclinic, *P-1*, *a* = 4.9858(7) Å, *b* = 7.2976(9) Å, *c* = 11.5769(13) Å, *α* = 86.779(9)°; *β* = 87.374(10)°; *γ* = 85.973(10)°; *V* = 419.14(9) Å^3^; *d*_calc_ = 1.632 gcm^−3^; *μ* = 1.470 mm^−1^; F(000) = 208; *θ* full: 67.7 ^o^; number of collected/independent/unique reflection (*R*_int_ = 1.40%) = 2592/1689/1467; no. of parameters/restrains = 133/2; GOF = 1.044; R[F]/wR[F] (I ≥ 3σ(I)) = 3.73%/9.92%; Δ*ρ*^max/min^ = +0.31/−0.21 eÅ^3^.

### 3.6. Theoretical Calculations 

To calculate the dimer interaction energies, a single-point calculations on M062X [36] 6–311 + G(d,p) [37] level of theory have been performed. The initial geometries were extracted from the experimental structures. The obtained values were corrected for the basis-set superposition error using the counterpoise procedure. At the same level of theory, a potential energy scan of the boronic group was performed with the step size 10°. The topological analyses of the calculated electron densities of all studied systems was prepared for the ground state optimized geometries at the same level of theory. It was accomplished in terms of the QTAIM approach and was carried out using AIMAll program [41]. In the framework of this approach, critical points (CPs) together with the bond paths (BPs) were found. The bond critical points (BCPs) were analyzed for intramolecular O-H…O HBs. The energy of the intramolecular HB interactions were estimated on the basis of empirical equations proposed by Espinosa, Abramov and Lecomte [38]. Within this approach, intermolecular interaction is considered to be equal to approximately half of the estimated potential energy density value at the critical point (V(rBCP)).

### 3.7. Biological Activity

The antibacterial activity of compounds was tested against Gram-positive bacteria—*Bacillus cereus* (CCM 2010) and Gram-negative bacteria—*Escherichia coli* (CCM 5172), obtained from the Czech Collection of Microorganisms (Masaryk University). Bacteria were routinely maintained at 34 °C on nutrient broth which consists of peptone (5 g) and meat extract (3 g) in 1000 mL of distilled water.

The activity of tested compounds was established using both the agar well diffusion method and by determining the minimal inhibitory concentration (MIC) values. In the case of the diffusion tests, 0.5mL of a 24-h culture containing 10^6^–10^7^ bacterial cells was uniformly spread out on the surface of the solidified nutrient agar medium and allowed to dry. In such prepared cultures, 3-mm wells were cut and filled with DMSO containing 100, 50, 25 or 10μg of the tested compound. As the controls, DMSO and water solutions of 50 μg of streptomycin were used. Cultures of bacteria were incubated for 24 h at 34 °C. The antimicrobial activity was estimated by the measurement of the inhibition zone of bacterial growth around the place of application of the tested substances. The experiments were performed in triplicate.

The minimal inhibitory concentration (MIC) was determined by a serial dilution method. For this purpose, 2 mL of bacterial cultures containing 10 ^6^ CFUs (colony forming units) per 1 mL of nutrient broth were prepared. The number of bacterial cells in medium was estimated using optical density measurements (OD) at an absorbance wavelength of 600 nm. To obtain the set of experimental concentrations of investigated compounds, the stock solution was serially diluted and placed to cultures, ensuring that the final concentration ranged from 1 to 500.0 µg  mL^−1^. Appropriate cultures were incubated for 24 h at 34°C. The experiments were performed in triplicate. The MIC was assessed based on the lowest concentration of tested compounds required to inhibit bacterial growth (detected as the lack of visible turbidity) and by spectrophotometry by measuring the OD_600_.

## 4. Conclusions

Compounds **1**–**3** were characterized by ^1^H, ^13^C, ^11^B and ^19^F-NMR spectroscopy. The commercial sample of **2** contained considerable amounts of the corresponding boroxine, whereas the acetone solution of **1** and **3** exclusively contained signals corresponding to boronic acids. The addition of a drop of D_2_O to its acetone solution resulted in shifting the equilibrium towards boronic acid as well as shifting all the signals in the NMR spectra. The introduction of the -OCF_3_ group influences the acidity, depending, however, on the position of a substituent with the *ortho* isomer being the least acidic. Typical hydrogen bonded dimers are the basic primary structural motives of compounds **1** and **3** in the solid state. Amongst the three isomeric (trifluoromethoxy)phenylboronic acids studied, compound **1**, containing an *ortho* substituent, displays an intramolecular hydrogen bond present in the solid state. However, due to a strong electron-withdrawing effect of the OCF_3_ substituent, that interaction is weaker than analogous one found in **1a**. Despite the promising results of docking studies, none of the studied compounds reveal considerable activity against *Escherichia coli*. At the same time, compounds **2** and **3** display limited activity against *Bacillus cereus* with MIC values of 125 µg/mL.

## Figures and Tables

**Table 2:** ^1^H-NMR chemical shifts (ppm) in acetone-d_6_, multiplicity and detected coupling constants in spectra of **1**–**3**.

	B(OH)_2_	H2	H3	H4	H5	H6
**1**	7.33 (s)	-	7.31–7.25 (m)	7.50 (ddd) *^3^J_H-H_* = 12.5 Hz *^3^J_H-H_* = 8.2 Hz *^4^J_H-H_* = 1.8 Hz	7.40–7.32 (m) overlapping with B(OH)_2_ signal *	7.76 (dd) *^3^J_H-H_* = 7.3 Hz *^4^J_H-H_* = 1.8 Hz
**2**	7.64 (s)	7.79–7.72 (m)	-	7.40–7.31 (m)	7.53–7.44 (m)	7.87 (ddd) *^3^J_H-H_* = 7.3 Hz *^4^J_H-H_* = 1.8 Hz *^4^J_H-H_* = 1 Hz
**3**	7.32 (s)	8.08–7.94 (m)	7.34–7.24 (m)	-	7.34–7.24 (m)	8.08–7.94 (m)

* In spectrum taken after the addition of a drop of D_2_O, the B(OH)_2_ signal shifted from 7.33 to 7.62 ppm and reduced its intensity, revealing the H5 signal as ddd; *^3^J_H-H_* = 12.5 Hz, *^3^J_H-H_* = 7.4 Hz, *^4^J_H-H_* = 1 Hz.

**Table 3:** ^13^C-NMR chemical shifts (ppm) in acetone-d_6_, multiplicity and detected coupling constants in spectra of **1**–**3**.

	OCF_3_	C1	C2	C3	C4	C5	C6
**1**	121.5 (q) *^1^J_C-F_* = 254.7 Hz	n.d.	153.3 (q) *^3^J_C-F_* = 1.7 Hz	121.5 (q) ^*4*^*J_C-F_* = 1.0 Hz	132.2 (s)	127.5 (s)	136.4 (s)
**2**	121.4 (q) *^1^J_C-F_* = 255.0 Hz	n.d.	126.8 (s)	149.7 (q) *^3^J_C-F_* = 1.7 Hz	123.6 (s)	130.2 (s)	133.7 (s)
**3**	121.4 (q) *^1^J_C-F_* = 255.5 Hz	n.d.	137.0 (s)	120.6 (s)	151.7 (q) *^3^J_C-F_* = 1.7 Hz	120.6 (s)	137.0 (s)

For α,α,α-trifluoro anisol in (CD_3_)_2_CO: 150.0(q, *^3^J_C-F_* = 1.9 Hz), 131.0 (s), 128.2 (s), 121.8 (s), 121.4 (q, *^1^J_C-F_* = 255.2 Hz).

**Table 4:** ^11^B and ^19^F-NMR chemical shifts in acetone-d_6_ (ppm).

	^11^B-NMR	^19^F-NMR
**1**	28 (s)	−56.93 (d), ^5^*J_H3-F_* = 1.4 Hz
**2**	28 (s)	−57.64 (dd), ^5^*J_H2-F_* = 0.9 Hz, ^5^*J_H4-F_* = 1 Hz
**3**	28 (s)	−57.59 (t) ^5^*J_H3H5-F_* = 1 Hz

**Table 5:** Basic geometrical parameters of molecules **1** and **3**.

	1	3-A *^a^*	3-B *^a^*
*d*_C1-B1_/Å	1.578(2)	1.569(4)	1.565(4)
*d*_O1-B1_/Å	1.365(2)	1.364(4)	1.359(3)
*d*_O2-B1_/Å	1.365(2)	1.358(3)	1.365(4)

*^a^* two molecules in asymmetric unit.

**Table 6:** Geometrical and electron density topological parameters characterizing intramolecular hydrogen bond interaction in **1** and **1a**. X-ray experimental values are provided in italics. Interaction energy values (*E*^EL^_int_) were calculated on the basis of the Espinosa–Lecomte estimation.

	$d_{O-H}$/Å	$d_{H\cdots O}$/Å	$d_{O\cdots O}/Å$	$\theta_{O-H\cdots O}/{}^{\circ}$	*ρ*(*r*_CP_)/e∙A^−3^	*L*(*r*_CP_) /e∙A^−5^	*E*^EL^_int_/kJ mol^−1^
**1**	0.961/*0.839*	2.081/*2.377*	2.817/*2.839(2)*	132.10/*115.3(1)*	0.136	0.471	19.0
**1a**	0.964/*0.859* [42]	1.924/*1.936*	2.719/*2.654*	138.10/*140.22*	0.177	0.655	29.3

**Table 7:** Second order perturbation theory analysis of Fock matrix in natural bond orbital (NBO) basis for selected Lewis orbitals in **1** and **1a**.

	Donor… Acceptor	Energy kJ mol^−1^
**1**	LP1(O) …BD3*(C-F1)	6.5
	LP1(O) …BD*(C_ar_-C_ar_)	4.8
	LP2(O) …BD1*(C-F2)	16.5
	LP2(O) …BD2*(C-F3)	15.1
**1a**	LP1(O) …BD3*(C-H1)	3.3
	LP1(O) …BD*(C_ar_-C_ar_)	6.6
	LP2(O) …BD1*(C-H2)	6.5
	LP2(O) …BD2*(C-H3)	6.4

* antibonding orbital.

**Table 8:** Properties of electron density around -OX (X = CF_3_, CH_3_) oxygen atom in **1** and **1a**.

	QTAIM			NBO			
	*L*(*r*) (3, −3)	*ρ*(***r***_CP_)/e∙A^−3^	*L*(***r***_CP_) /e∙A^−5^	Orbital	Occupancy/*e*	Orbital	Occupancy/*e*
**1**	CC1(O)	6.468	125.5	LP1(O)	1.900	BD*(O–H)	0.011
	CC2 (O)	6.379	125.8	LP2(O)	1.935	BD1*(C–F) BD2*(C–F) BD3*(C–F)	0.111 0.107 0.091
**1a**	CC1 (O)	6.479	121.3	LP1(O)	1.868	BD*(O–H)	0.016
	CC2 (O)	6.379	121.2	LP2(O)	1.957	BD1*(C–H) BD2*(C–H) BD3*(C–H)	0.017 0.017 0.008

* antibonding orbital.

**Table 9:** Chosen results of docking studies of **1**–**3** with LeuRS of *Escherichia coli*.

	Lowest Binding Energy (kcal mol^−1^)	Mean Binding Energy (kcal mol^−1^)	Number in Cluster	Inhibition Constant (μM)	Number of H-Bonds
**1**	−4.65	−4.45	73	391.52	4
**2**	−4.73	−4.59	36	341.78	3
**3**	−5.29	−4.92	94	132.74	5
**1-AMP**	−7.32	−6.91	9	4.30	2
**2-AMP**	−7.07	−6.77	6	6.62	1
**3-AMP**	−6.84	−6.14	22	9.70	3

**Table 10:** Results of anti *E. coli* activity studies of compounds **1**–**3** compared to reference probes; zones of growth inhibition are given in (mm). MIC: minimal inhibitory concentration.

	10 μg	25 μg	50 μg	100 μg	DMSO	MIC (μg/mL)
**1**	0	0	0	0	0	>500
**2**	0	0	0	1.7 ± 1.5	0	250
**3**	0	0	0	1.3 ± 0.6	0	250
Streptomycin	13 ± 1	14.7 ± 0.6	15.7 ± 0.6	16 ± 0	0	2

**Table 11:** Results of anti *B. cereus* activity studies of compounds **1**–**3** compared to reference probes; zones of growth inhibition are given in (mm). MIC: minimal inhibitory concentration.

	10 μg	25 μg	50 μg	100 μg	DMSO	MIC (μg/mL)
**1**	0	0	0	0	0	>500
**2**	0	0	6 ± 1	11 ± 1	0	125
**3**	0	5 ± 2	8 ± 1	12 ± 1	0	125
Streptomycin	16 ± 1	19 ± 1	20 ± 1	23 ± 1	0	8

## Data Availability

Not applicable.

## Supplementary Materials

The following are available online. Figures S1–S12 titration curves and UV-VIS spectra, Figures S13–S24 NMR spectra.
