# Supplementary material for: (Trifluoromethoxy)Phenylboronic Acids: Structures, Properties, and Antibacterial Activity
Source: Molecules. 2021 Apr 1;26(7):2007. doi: 10.3390/molecules26072007 (PMC8036725; doi:10.3390/molecules26072007)
Supplement: Supplementary file 1 [file molecules-26-02007-s001.pdf]

(Trifluoromethoxy)phenylboronic acids: structures, properties, and antibacterial activity

Supplementary data

Agnieszka Adamczyk-Woźniak[a*], Jan T. Gozdalik[a], Ewa Kaczorowska[a], Krzysztof Durka[a], Dorota Wieczorek [b], Dorota Zarzeczańska [c], Andrzej Sporzyński [a,d]

[a] Faculty of Chemistry, Warsaw University of Technology, Noakowskiego 3, 00-664 Warsaw, Poland

[b] Faculty of Chemistry, University of Opole, Oleska 48, 45-052 Opole, Poland

[c] Faculty of Chemistry, University of Gdańsk, Wita Stwosza 63, 80-308 Gdańsk, Poland

[d] Faculty of Agriculture and Forestry, University of Warmia and Mazury, Oczapowskiego 8, 10-719 Olsztyn, Poland

*corresponding author: agnieszka@ch.pw.edu.pl

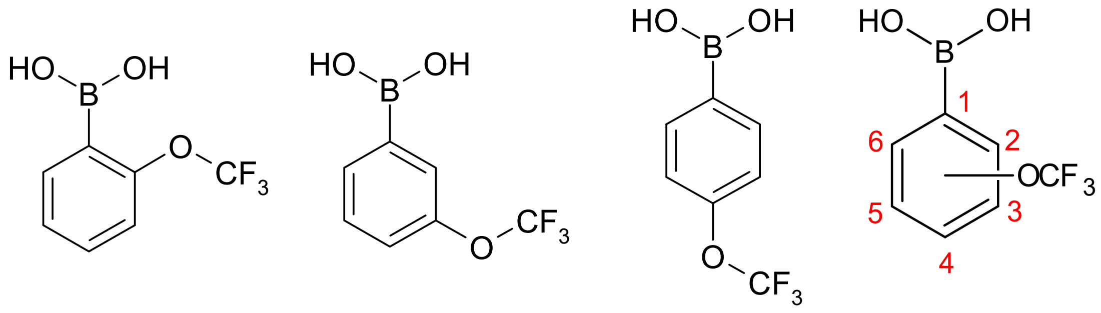

1 **2** **3**

Figure 1. Structure of investigated compounds (**1**–**3**) and atom numbering scheme.

1. Spectrophotometric titration

1.1. Compound **1**

Concentration of compound **1**: c=8.29·10^{-4} M

Concentration of NaOH: c=0.0409 M

Ionic strength: 0.1 M KCl

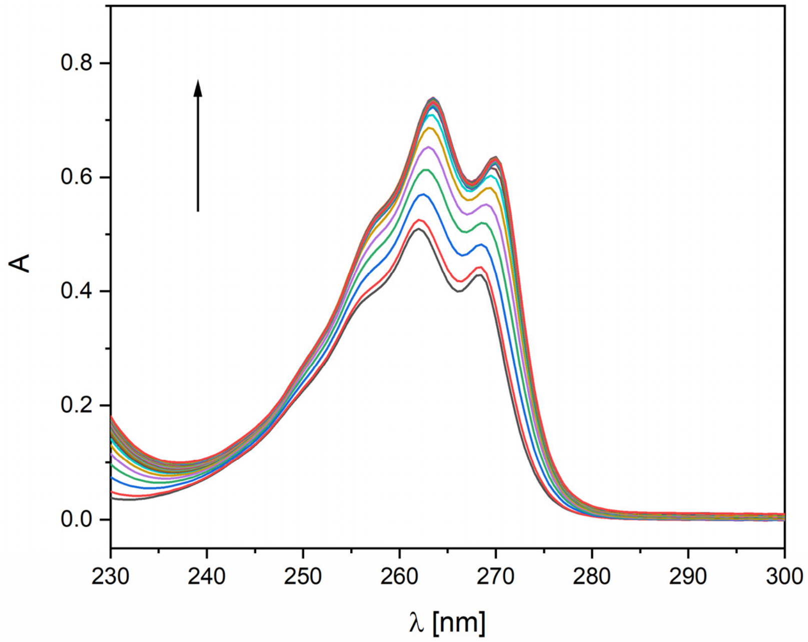

Figure S1. Spectrophotometric titration in pH range 6-12 in water (a correction to the dilution was taken into account). Arrows indicate changes in the absorbance with respect to the increase of pH.

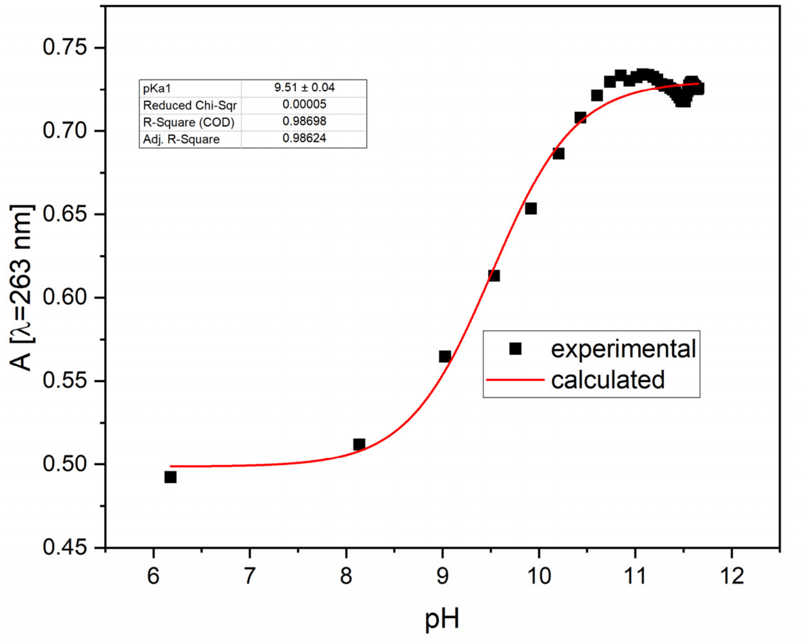

Figure S2. Analysis of spectroscopic data: absorbance change at 263 nm versus pH.

1.2. Compound **2**
Concentration of compound **2**: c=2.00·10^{-3} M
Concentration of NaOH: c=0.0409 M
Ionic strength: 0.1 M KCl

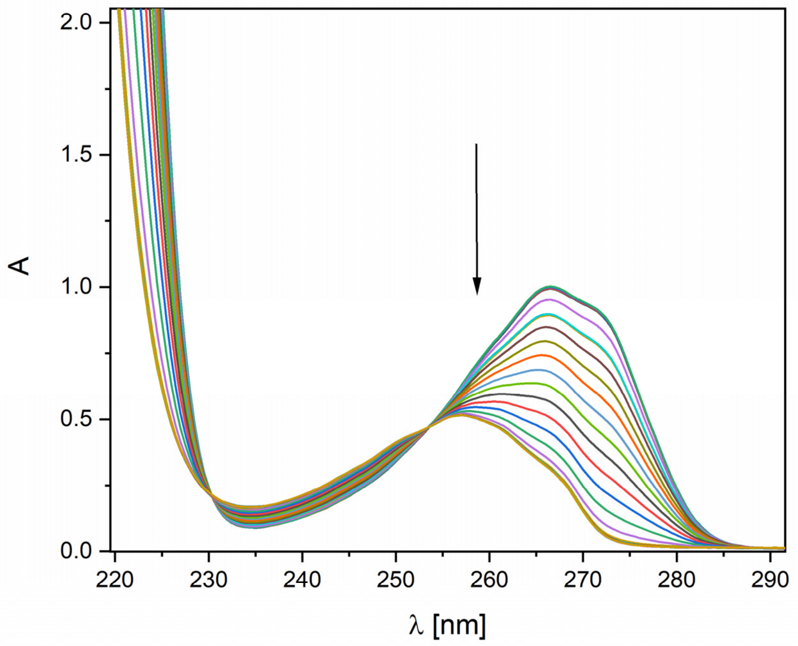

Figure S3. Spectrophotometric titration in pH range 6-11 in water (a correction to the dilution was taken into account). Arrows indicate changes in the absorbance with respect to the increase of pH.

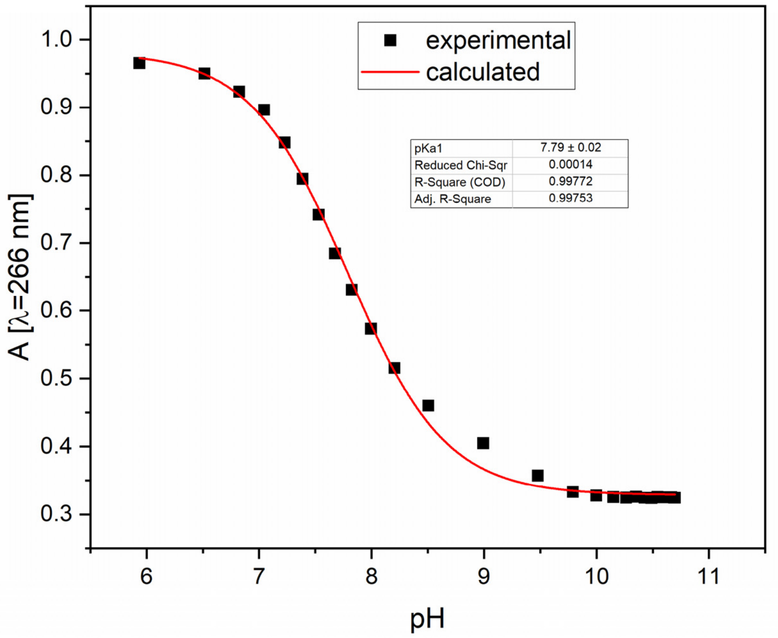

Figure S4. Analysis of spectroscopic data: absorbance change at 266 nm versus pH.

1.3. Compound **3**
Concentration of compound **3**: $c=1.22\cdot10^{-3}$ M
Concentration of NaOH: c=0.0409 M
Ionic strength: 0.1 M KCl

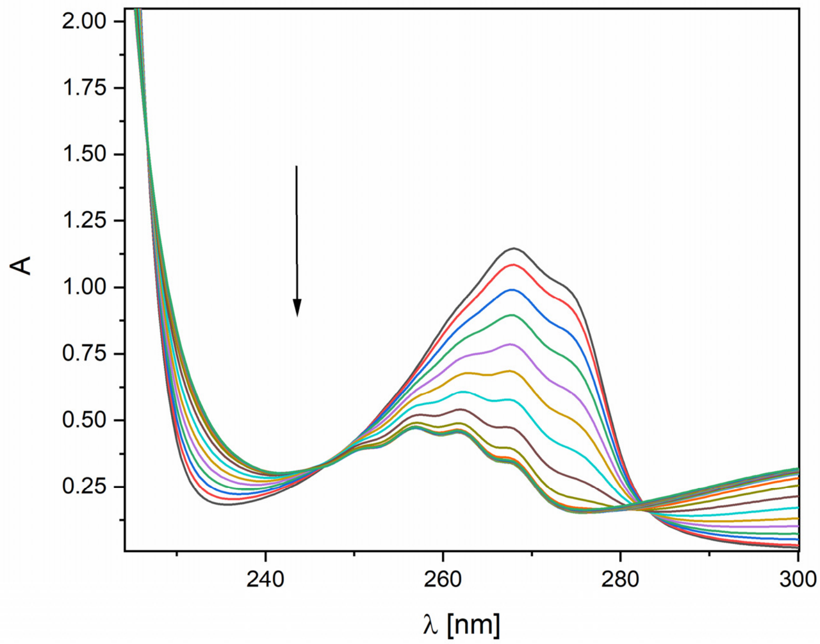

Figure S5. Spectrophotometric titration **3** in pH range 6-12 in water (a correction to the dilution was taken into account). Arrows indicate changes in the absorbance with respect to the increase of pH.

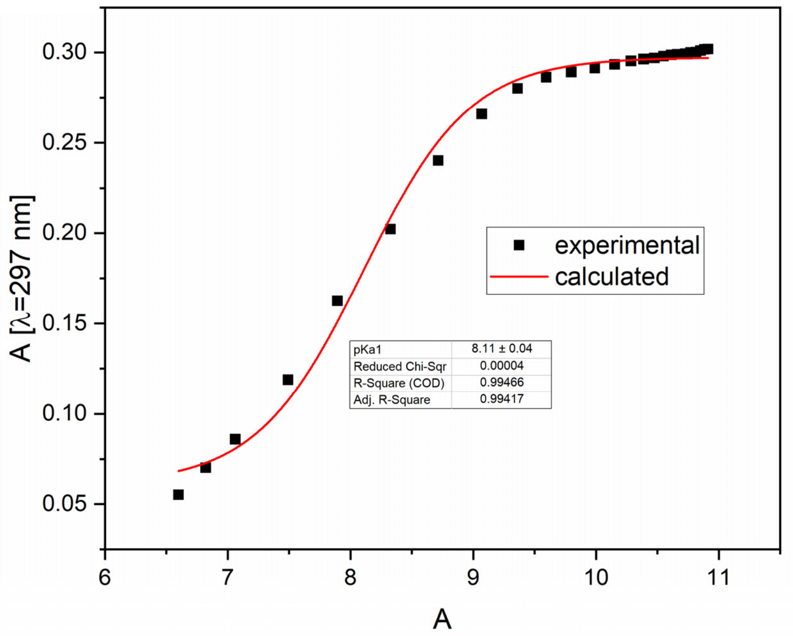

Figure S6. Analysis of spectroscopic data: absorbance change at 297 nm versus pH.

2. Potentiometric titration

2.1. Compound **1**
Concentration of compound **1**: $c=2.15\cdot10^{-3}$ M
Concentration of NaOH: c=0.0409 M
Ionic strength: 0.1 M KCl

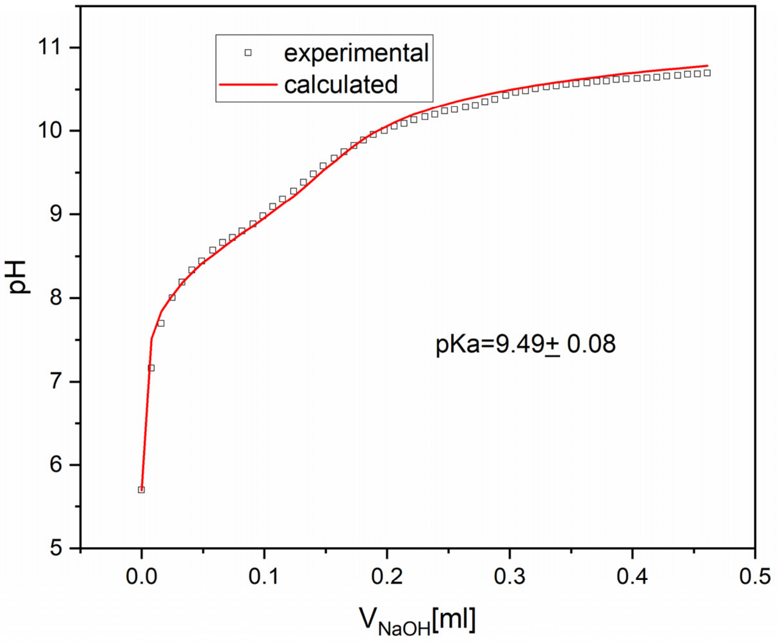

Figure S7. Potentiometric titration **1** with fitting of calculated data.

2.2. Compound **2**
Concentration of compound **1**: c=2.00·10^{-3} M
Concentration of NaOH: c=0.0409 M
Ionic strength: 0.1 M KCl

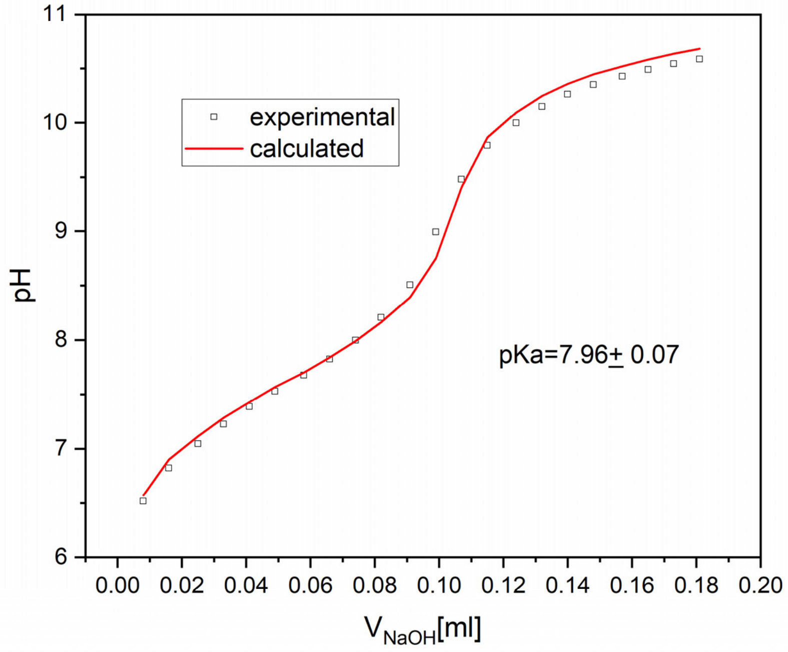

Figure S8. Potentiometric titration **2** with fitting of calculated data.

2.3. Compound **3**
Concentration of compound **3**: $c=1.22 \cdot 10^{-3}$ M
Concentration of NaOH: c=0.0409 M
Ionic strength: 0.1 M KCl

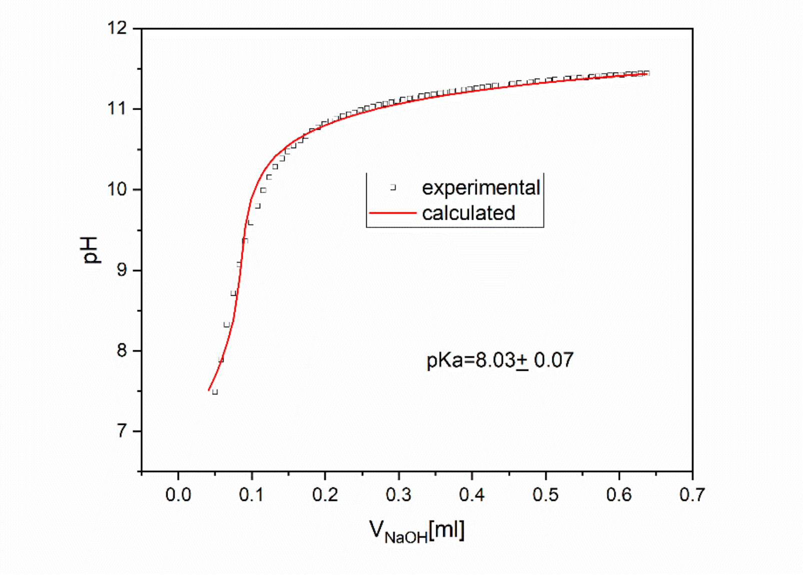

Figure S9. Potentiometric titration **3** with fitting of calculated data

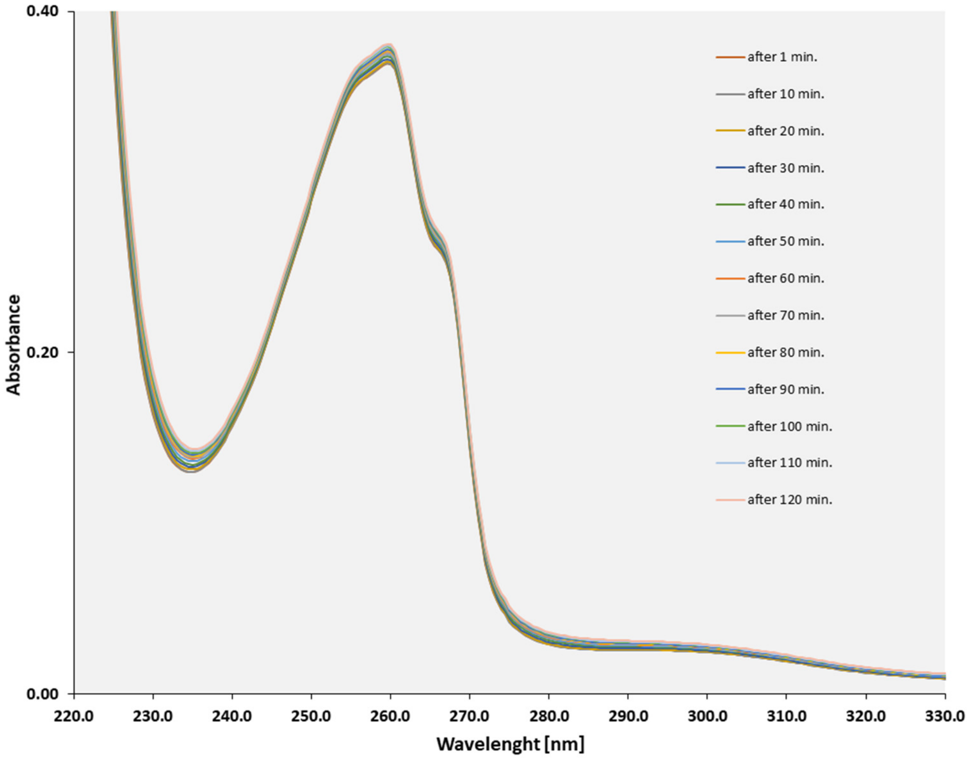

Figure S10. UV-Vis spectra of **1** solution in 0.025 M NaOH measured for 2 h after dissolution of the compound at room temperature.

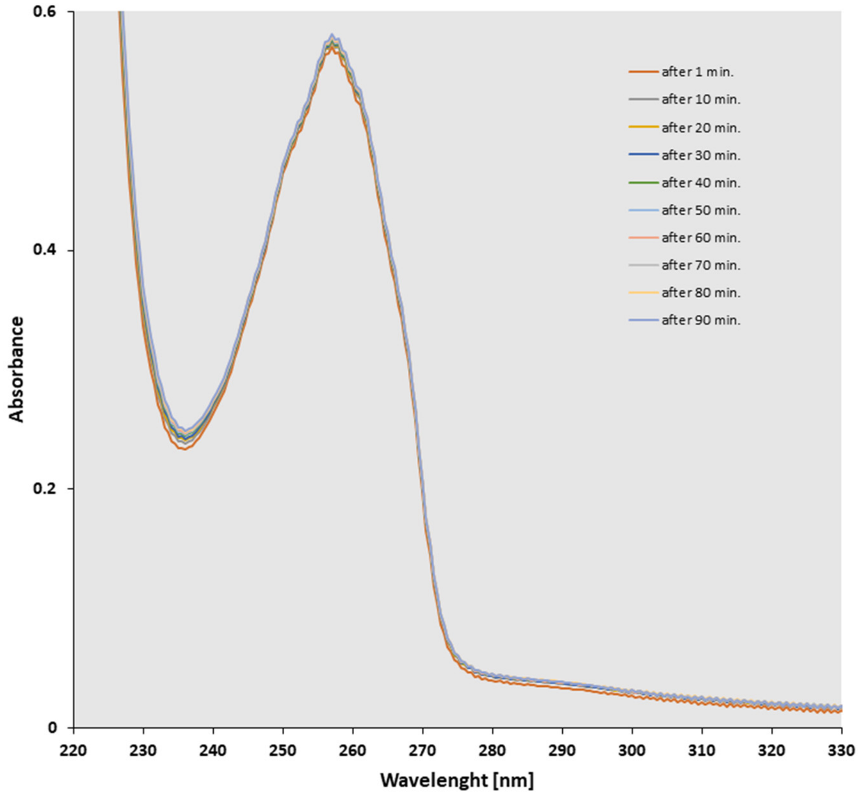

Figure S11. UV-Vis spectra of **2** solution in 0.025 M NaOH measured for 1.5 h after dissolution of the compound at room temperature.

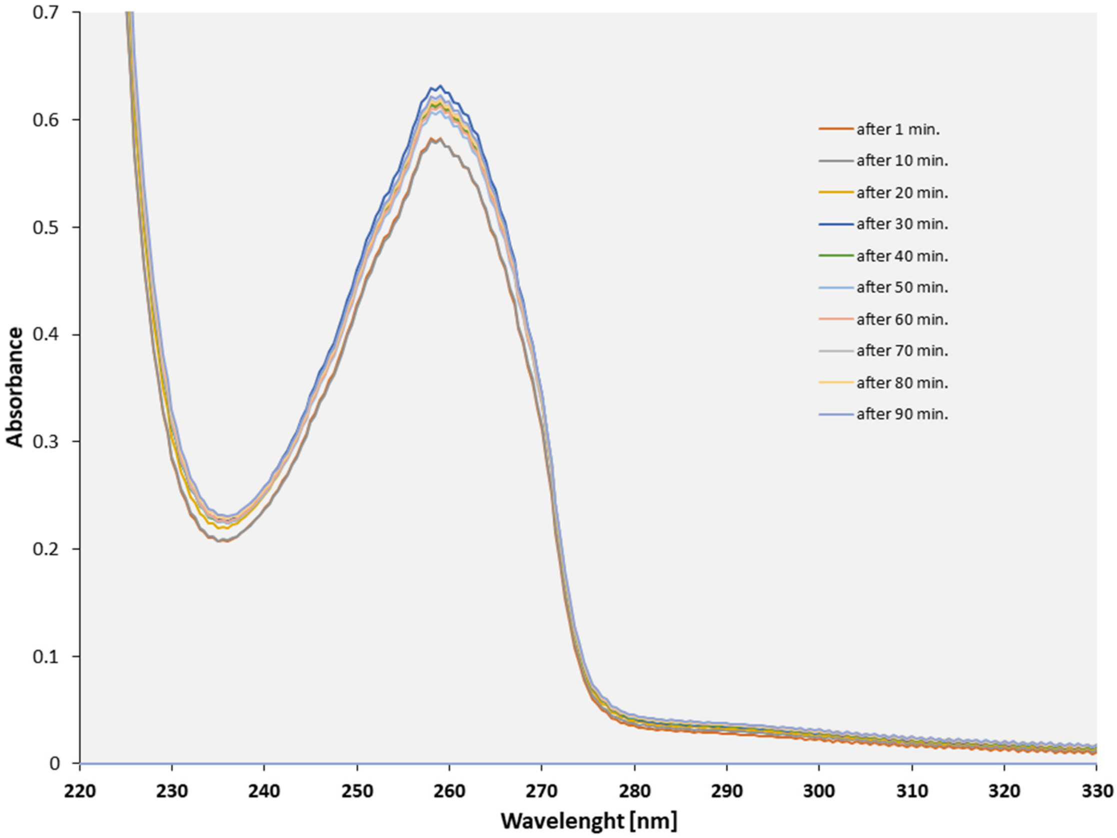

Figure S12. UV-Vis spectra of **3** solution in 0.025 M NaOH measured for 1.5 h after dissolution of the compound at room temperature.

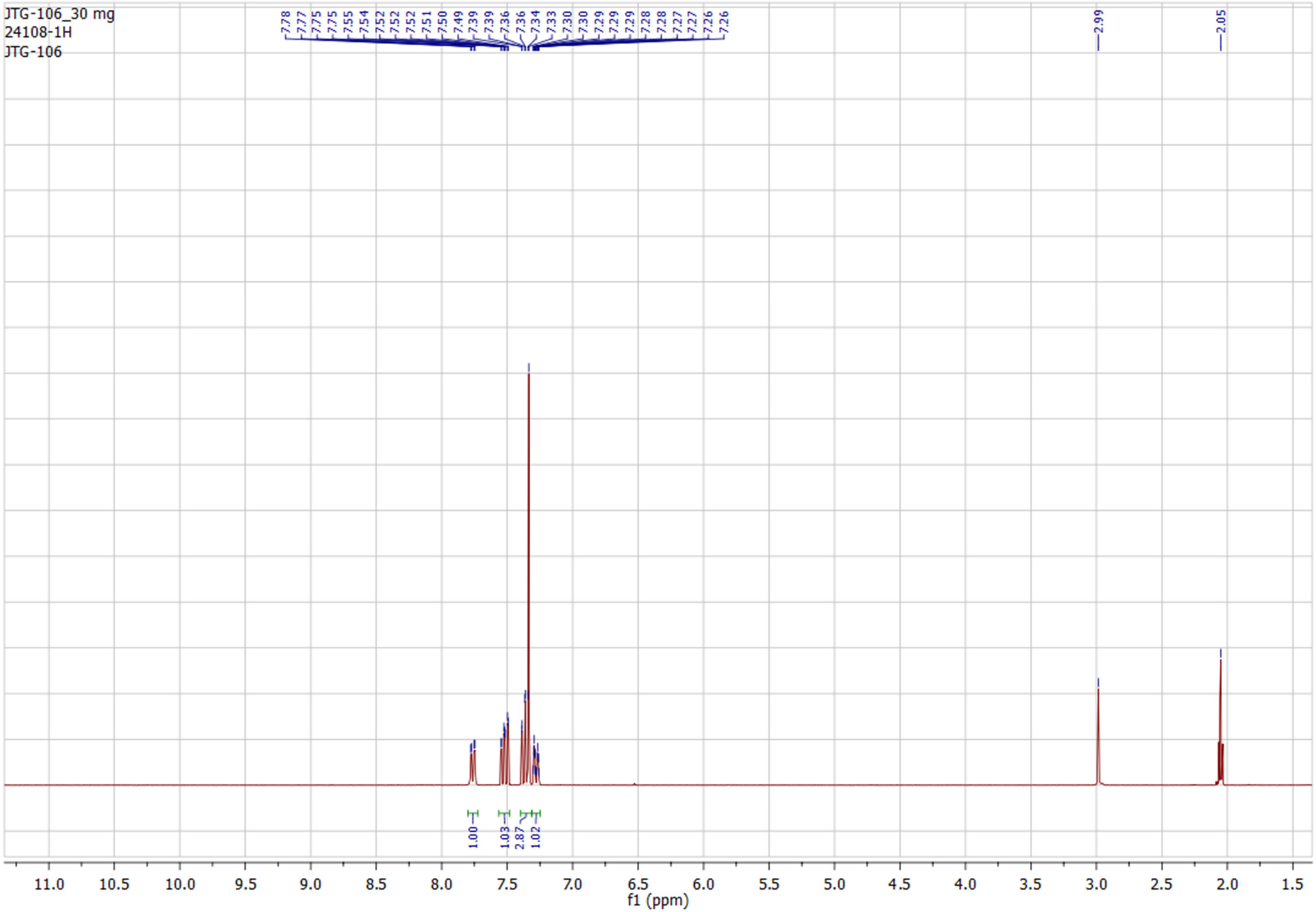

Figure S13. ^{1}H-NMR spectrum of **1** in $(CD_3)_2CO$ (full).

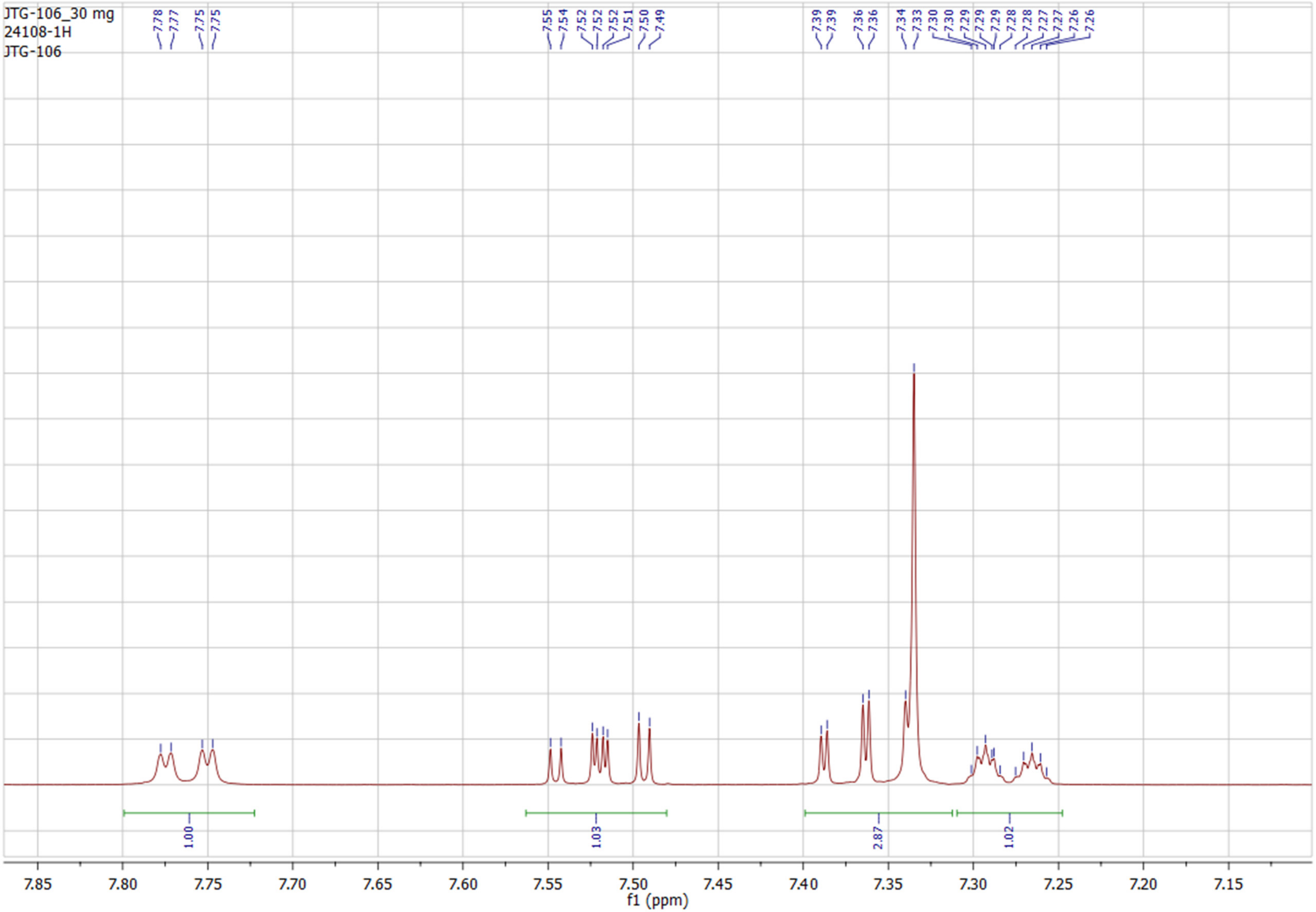

Figure S14. ^{1}H-NMR spectrum of **1** in $(CD_3)_2CO$ (expansion).

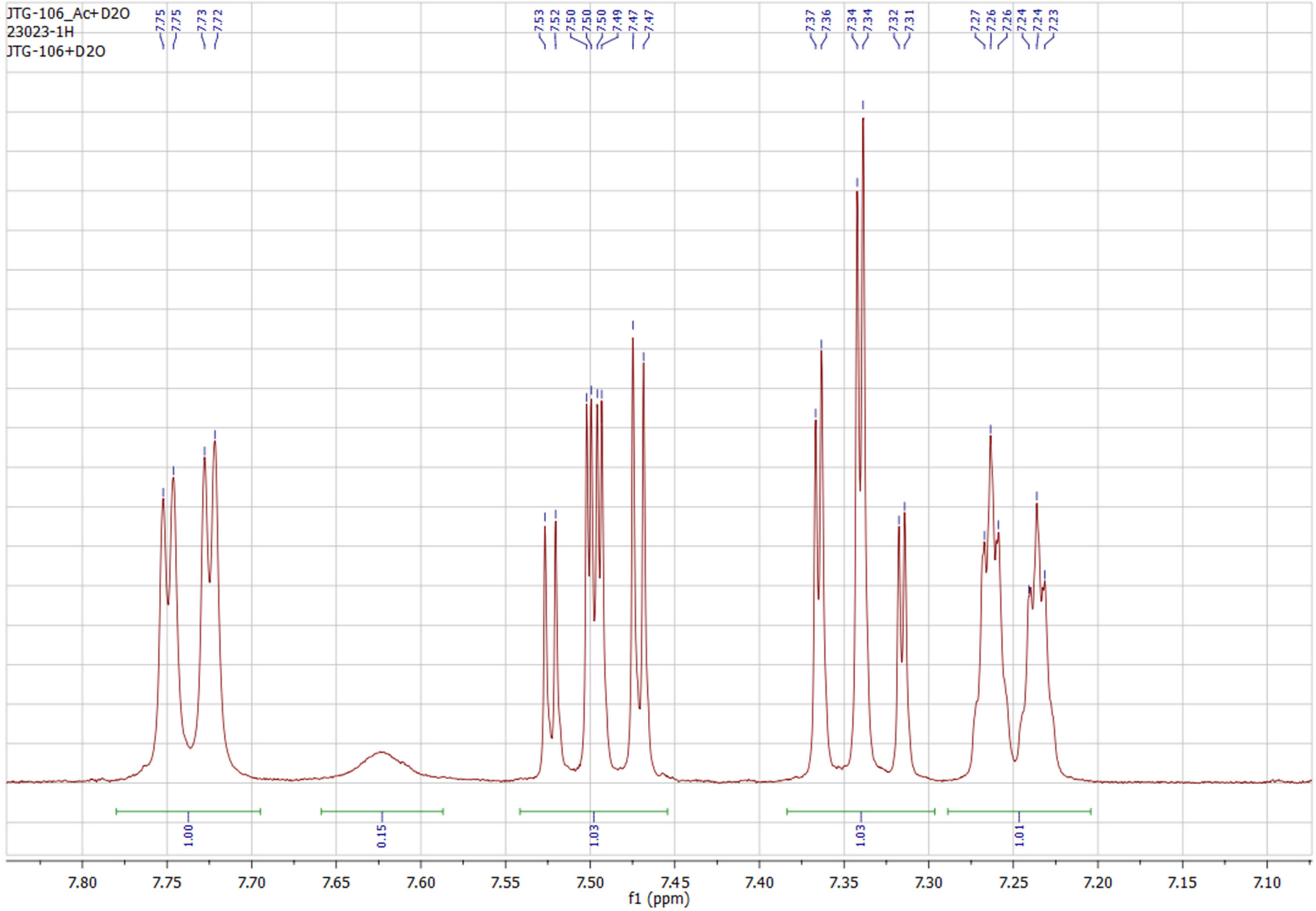

Figure S15. ^{1}H-NMR spectrum of **1** in $(CD_3)_2CO + D_2O$ (expansion).

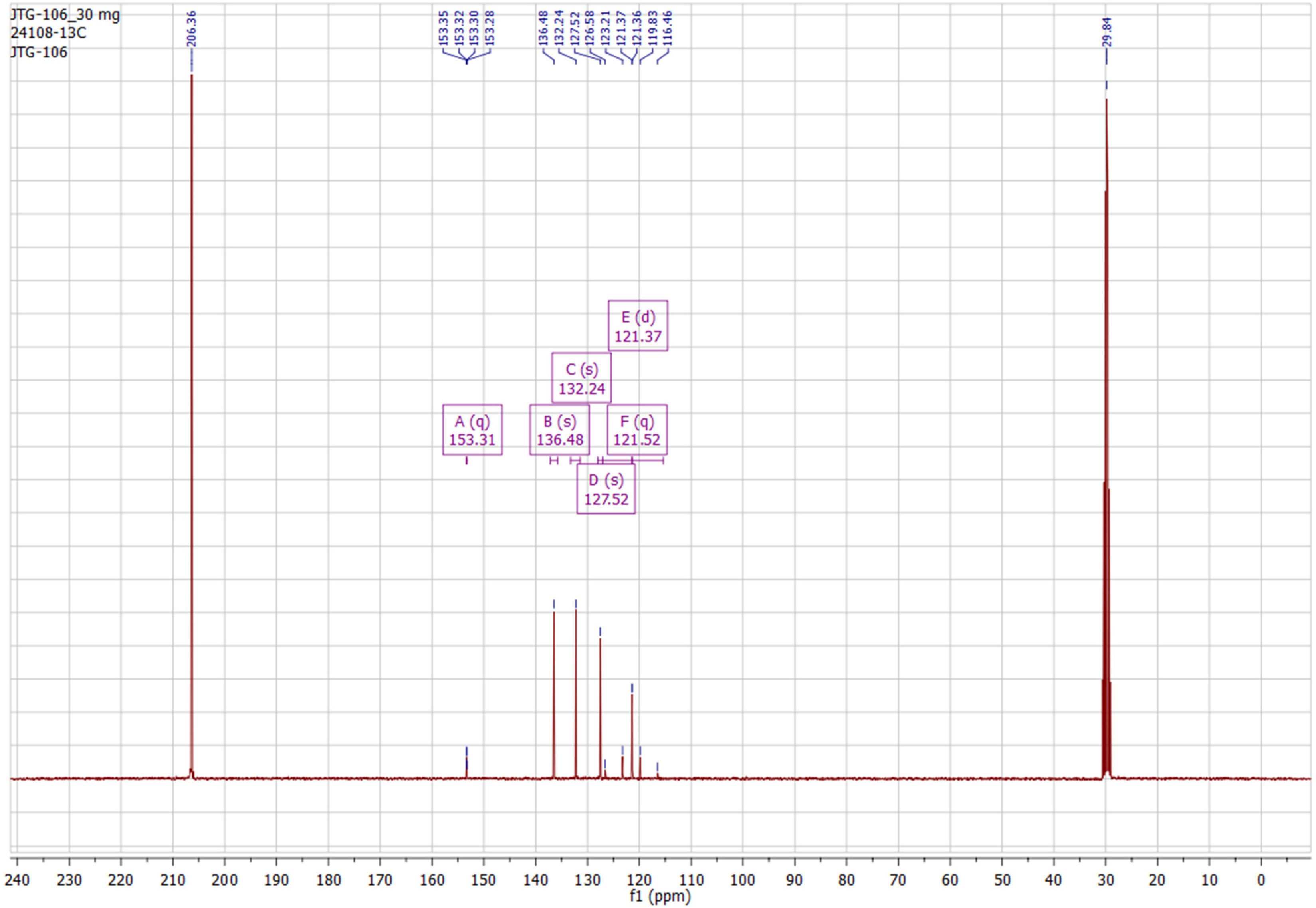

Figure S16. ^{13}C-NMR spectrum of **1** in $(CD_3)_2CO$ (full).

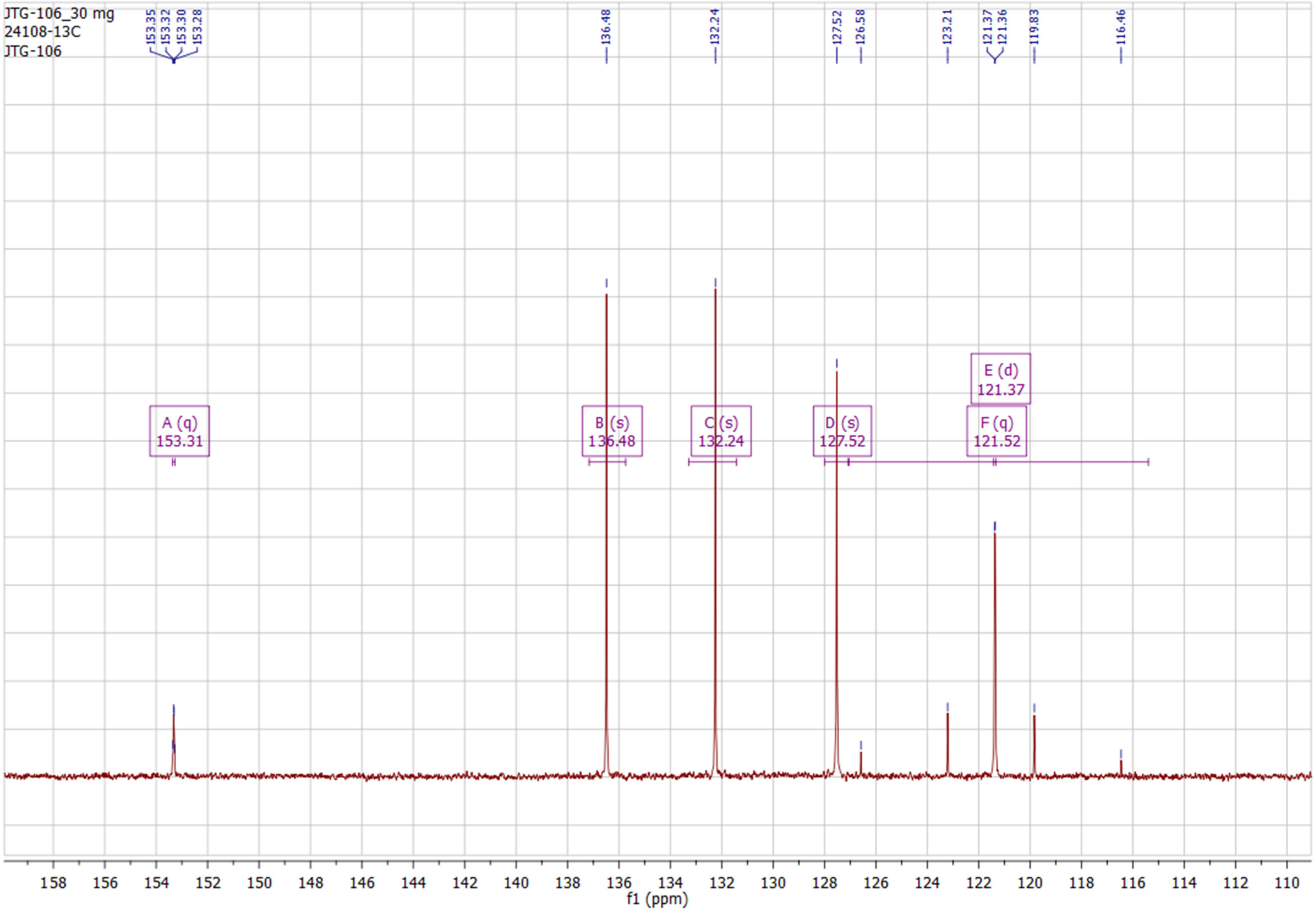

Figure S17. ^{13}C-NMR spectrum of **1** in $(CD_3)_2CO$ (expansion).

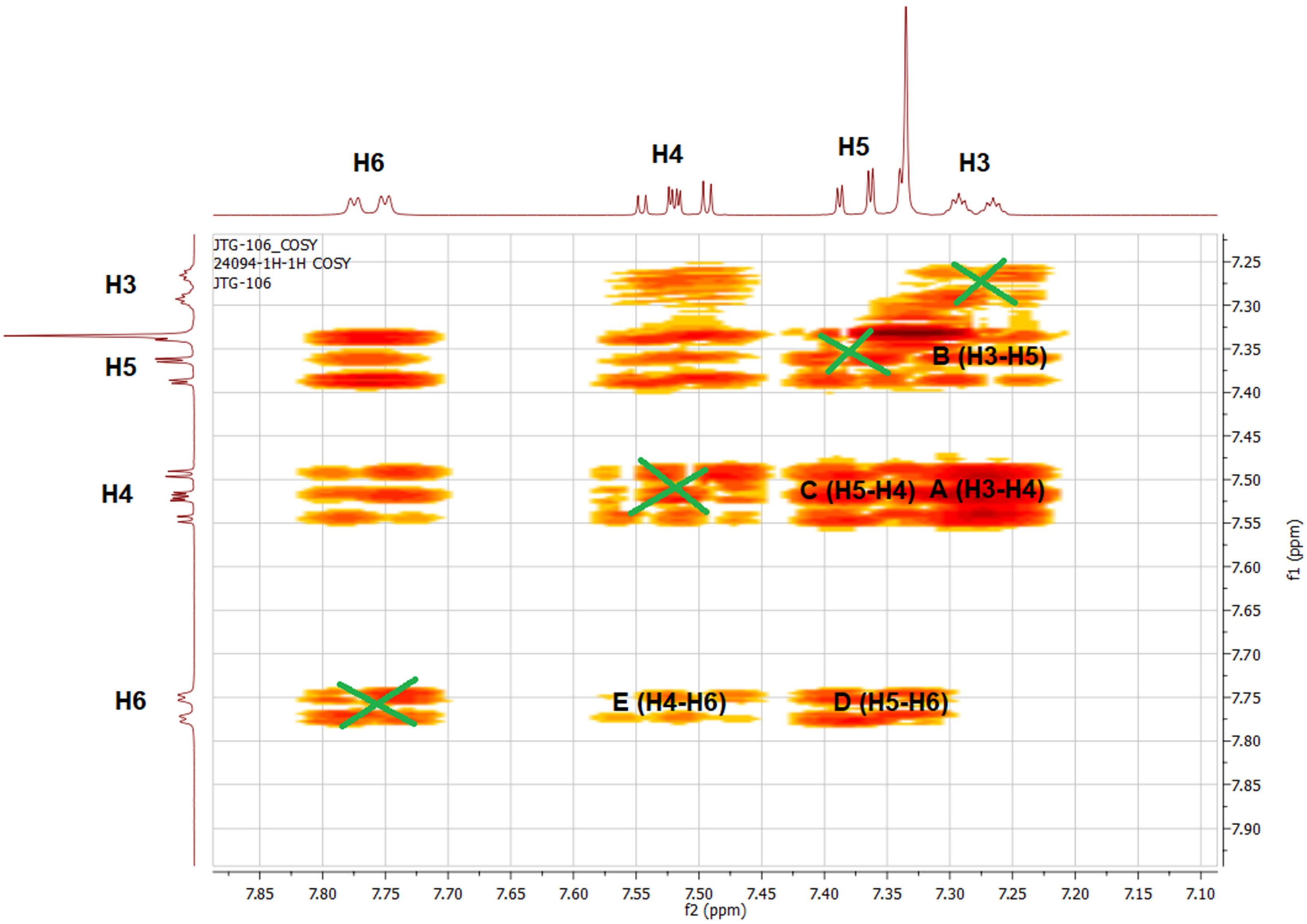

Figure S18. ^{1}H, ^{1}H-COSY spectrum of **1** in $(CD_3)_2CO$.

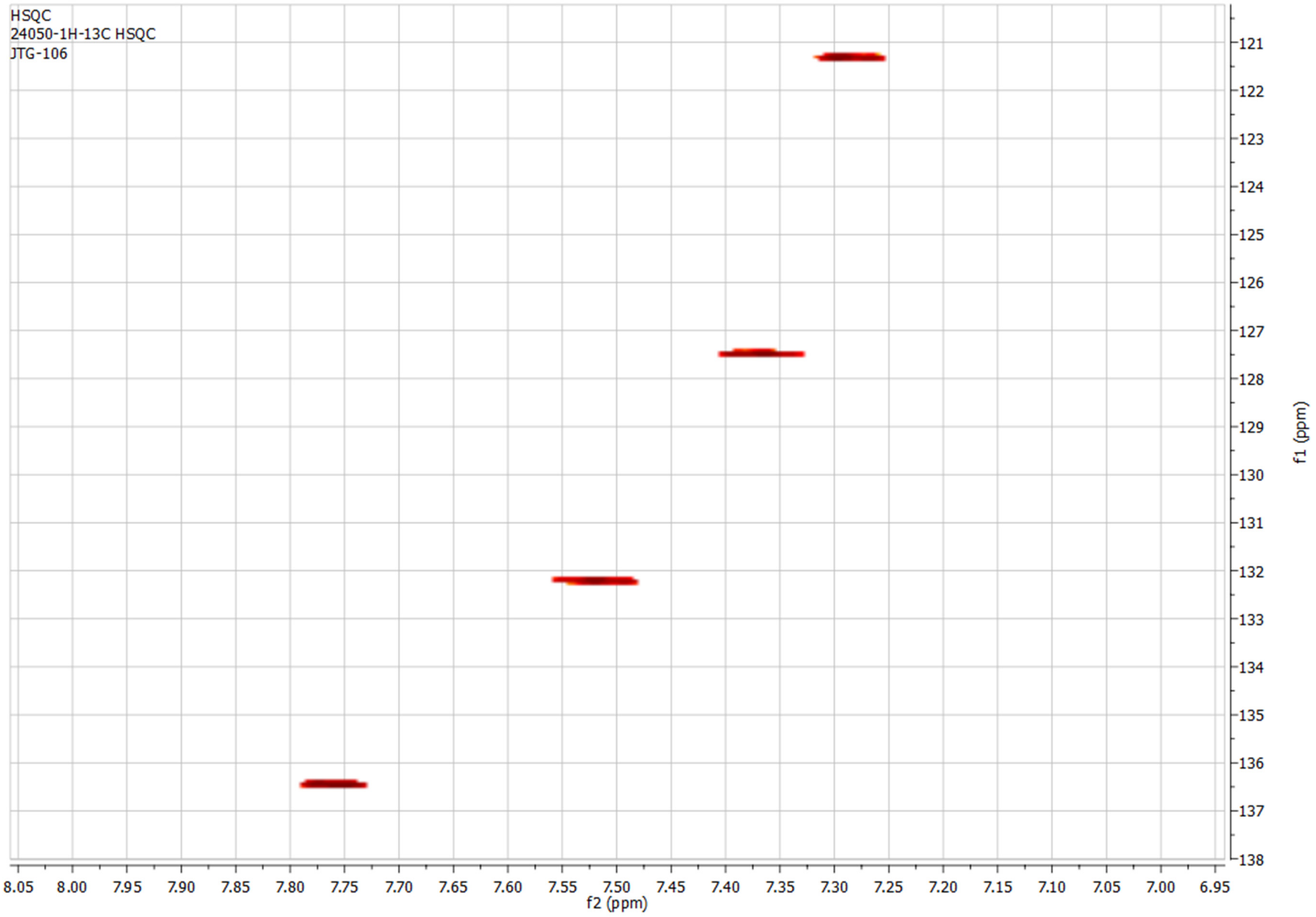

Figure S19. HSQC spectrum of **1** in $(CD_3)_2CO$.

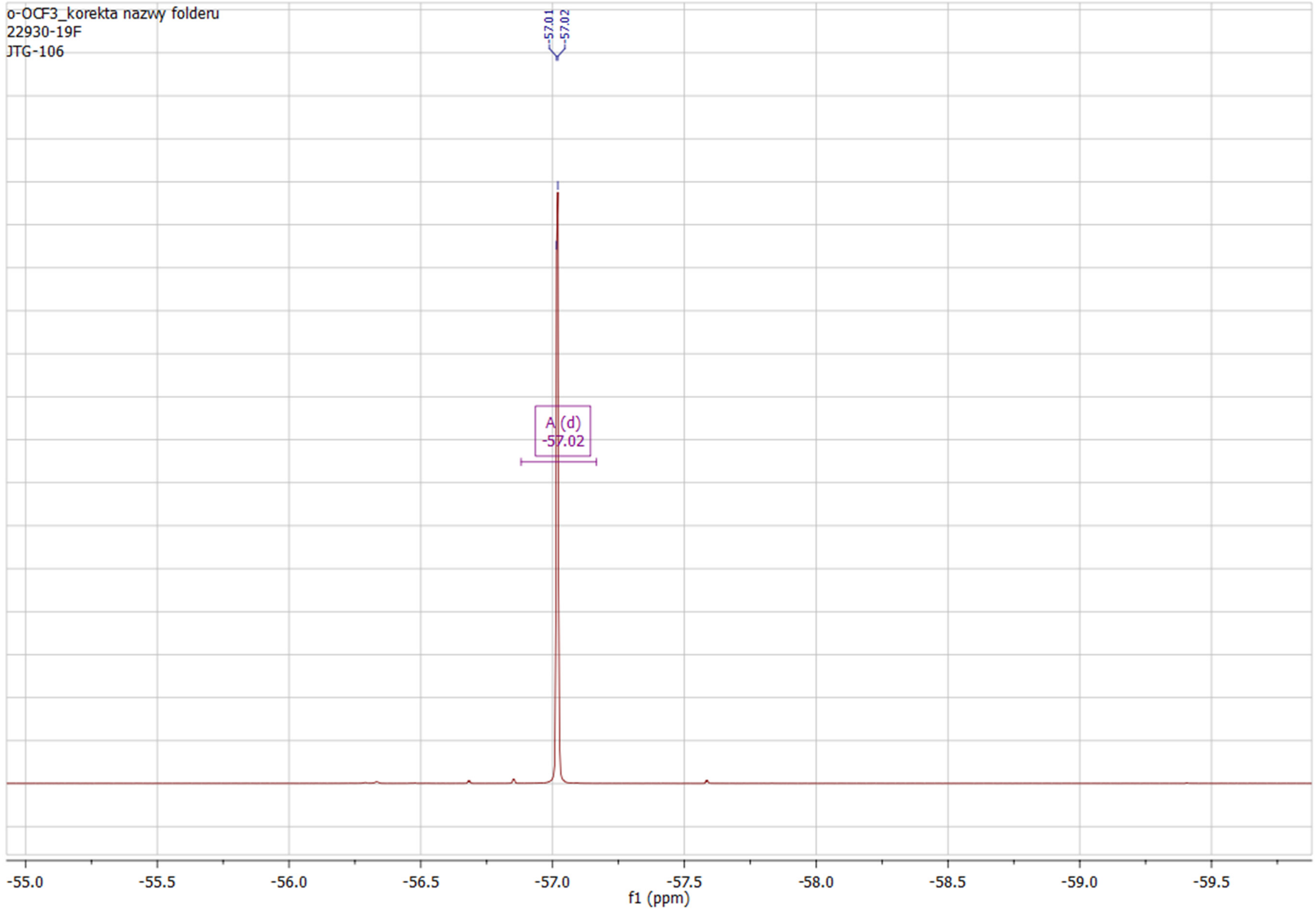

Figure S20. ^{19}FNMR spectrum of **1** in $(CD_3)_2CO$.

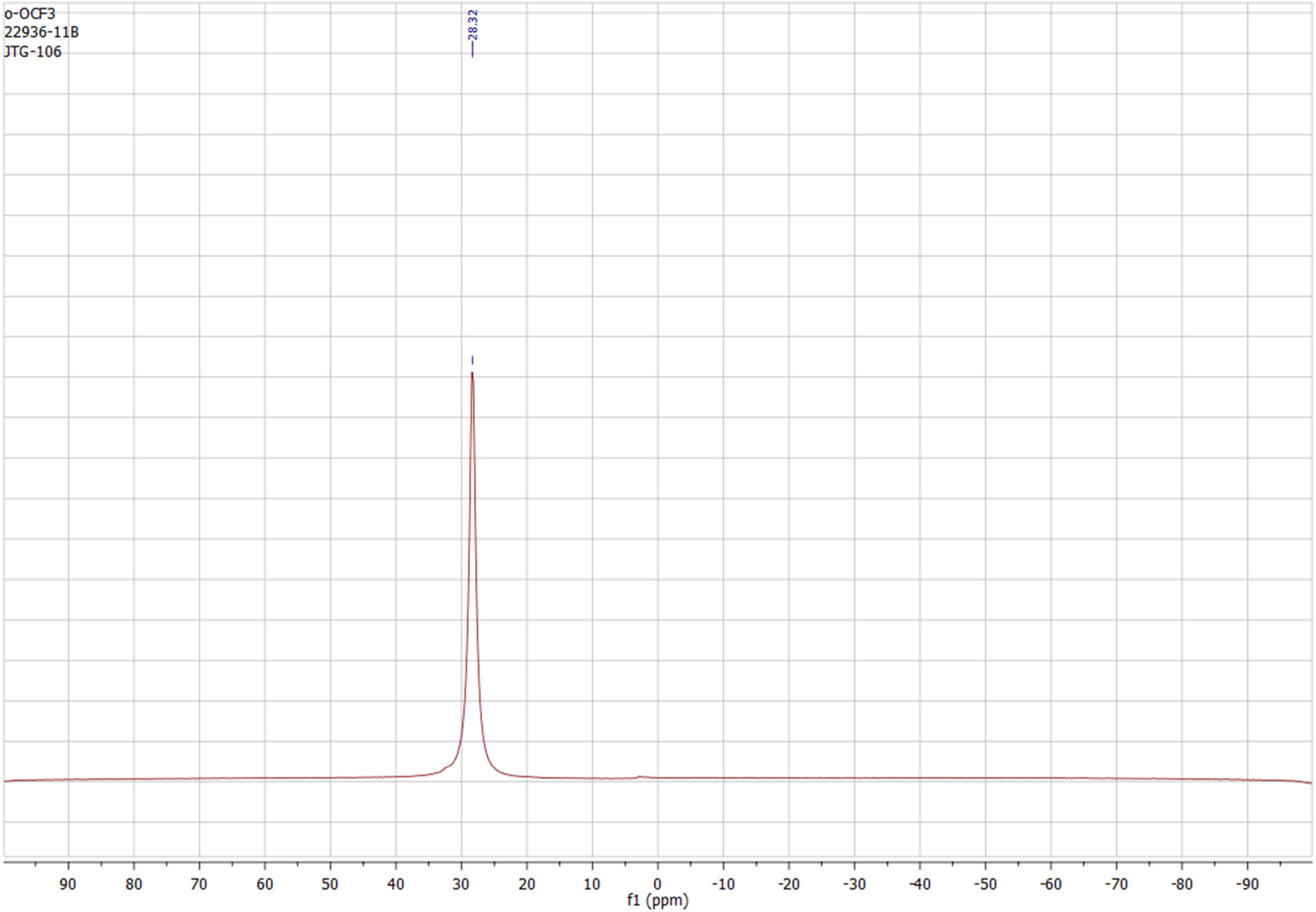

Figure S21. ^{11}B-NMR spectrum of **1** in $(CD_3)_2CO$.

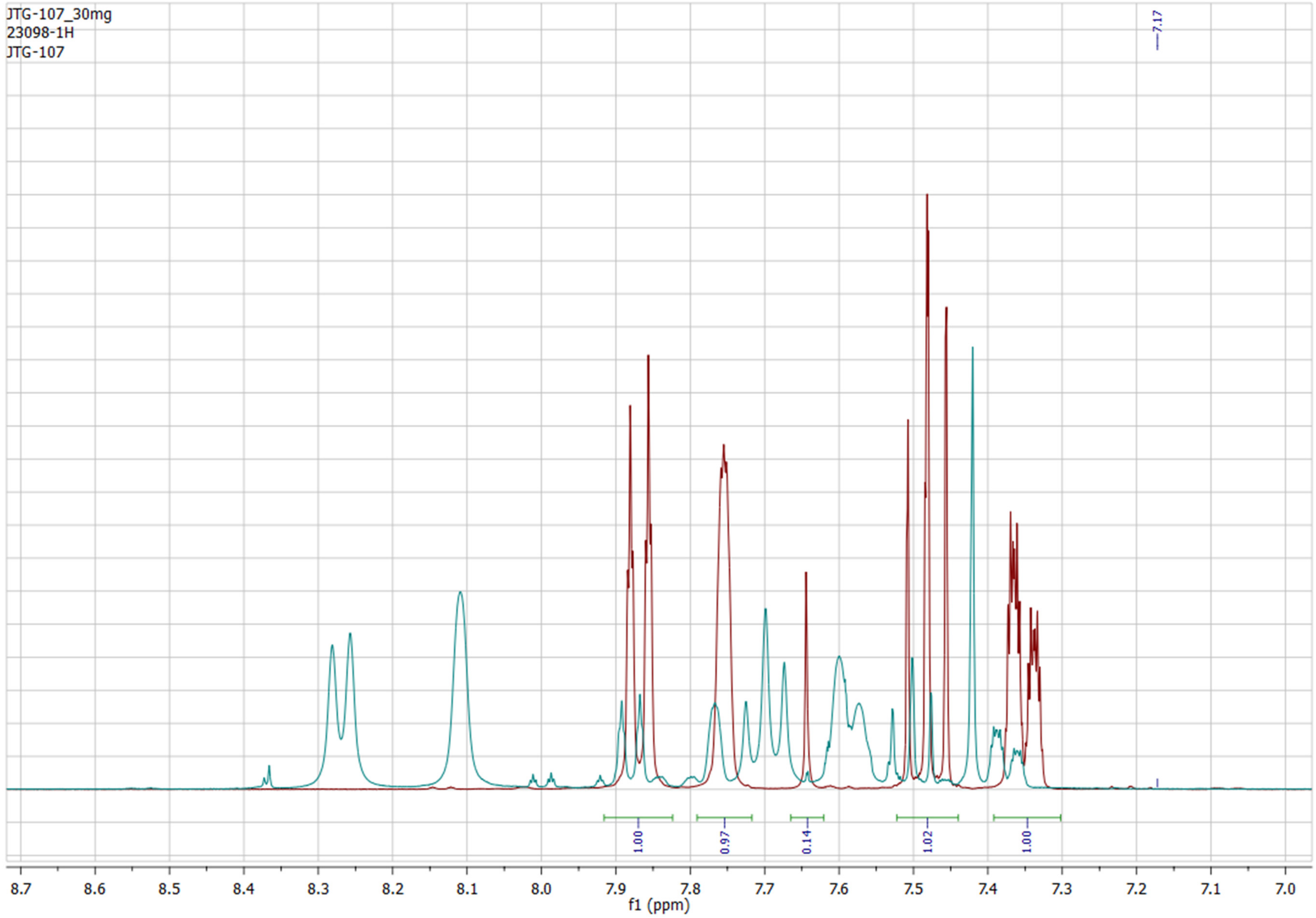

Figure S22. ^{1}H-NMR spectra of on-shelf sample **2** in $(CD_3)_2CO$ (blue) and in $(CD_3)_2CO$ + a drop of D_2O(red).

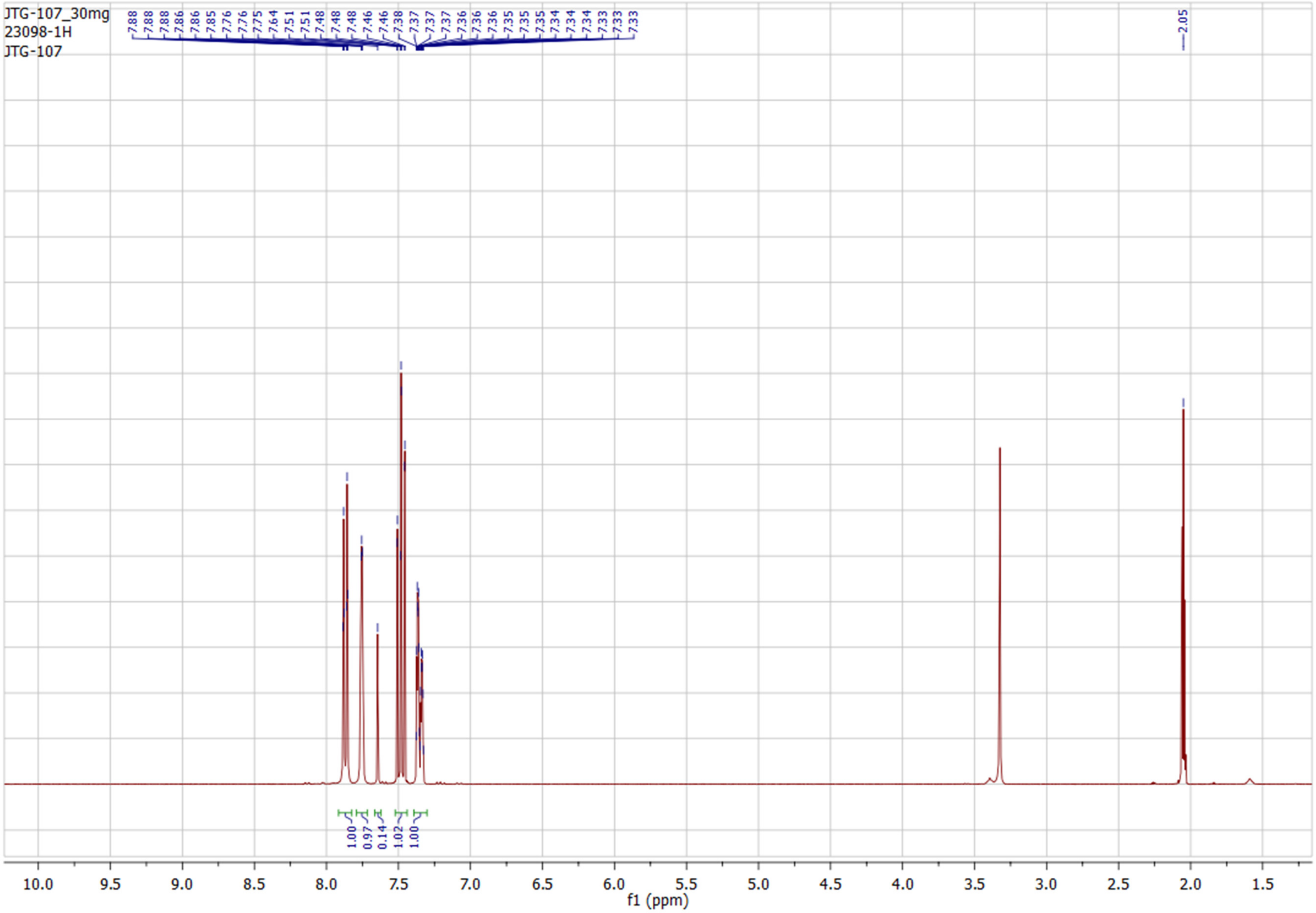

Figure S23. ^{1}H-NMR spectra of **2** in $(CD_3)_2CO$ + a drop of D_2O (full spectrum).

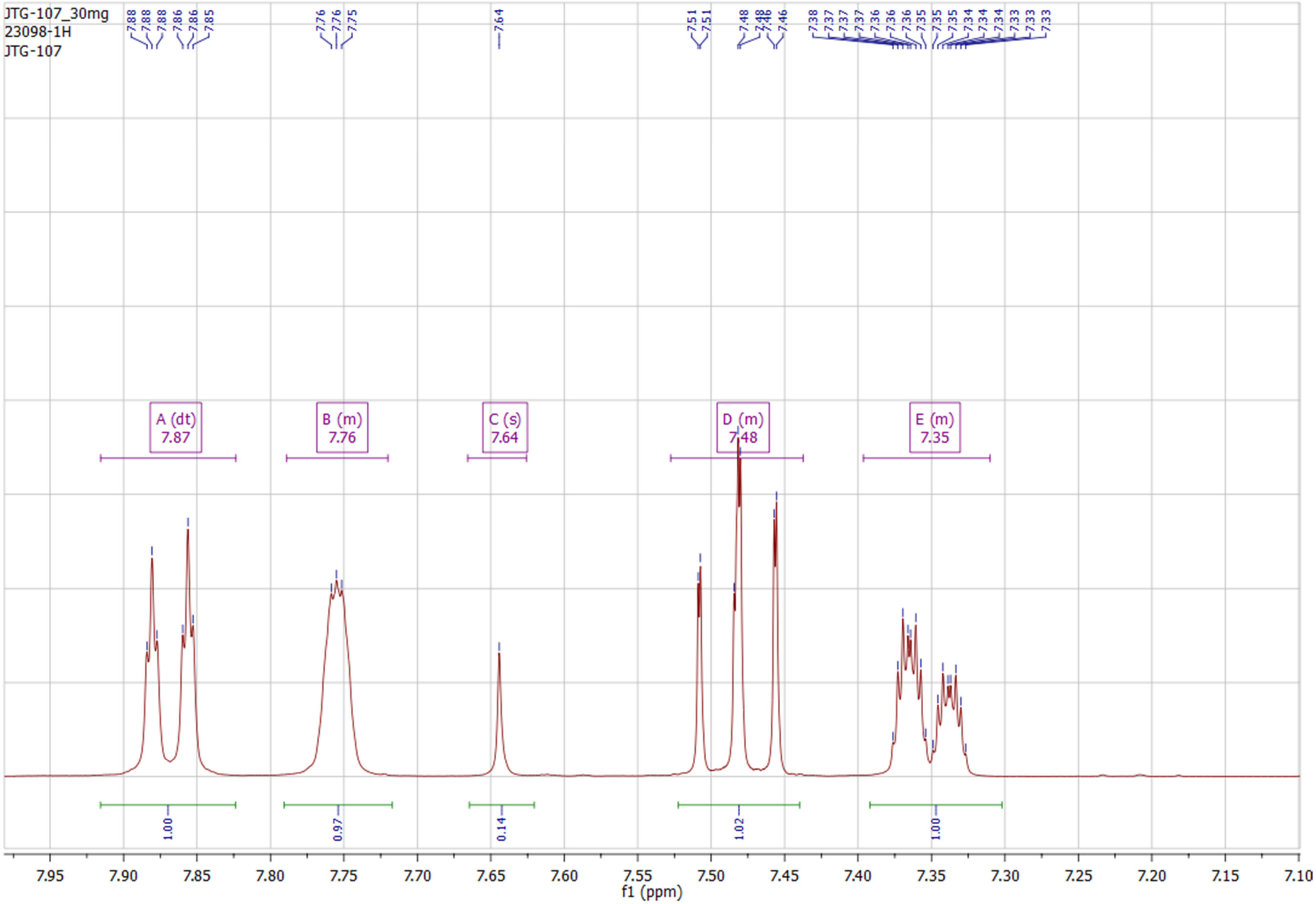

Figure S24. ^{1}H-NMR spectrum of **2** in $(CD_3)_2CO$ + a drop of D_2O (expansion).

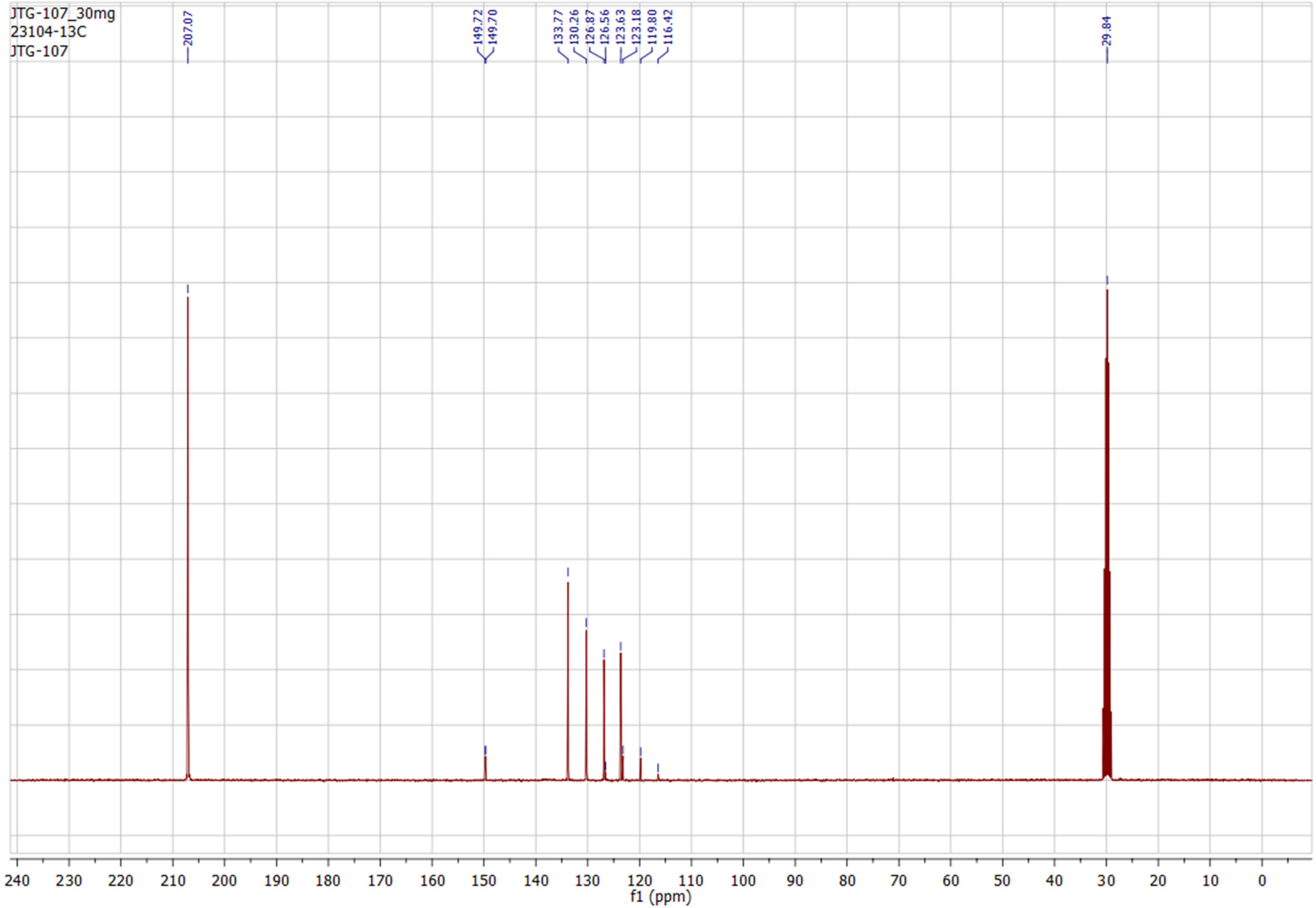

Figure S25. ^{13}C-NMR spectrum of **2** in $(CD_3)_2CO$ + a drop of D_2O (full).

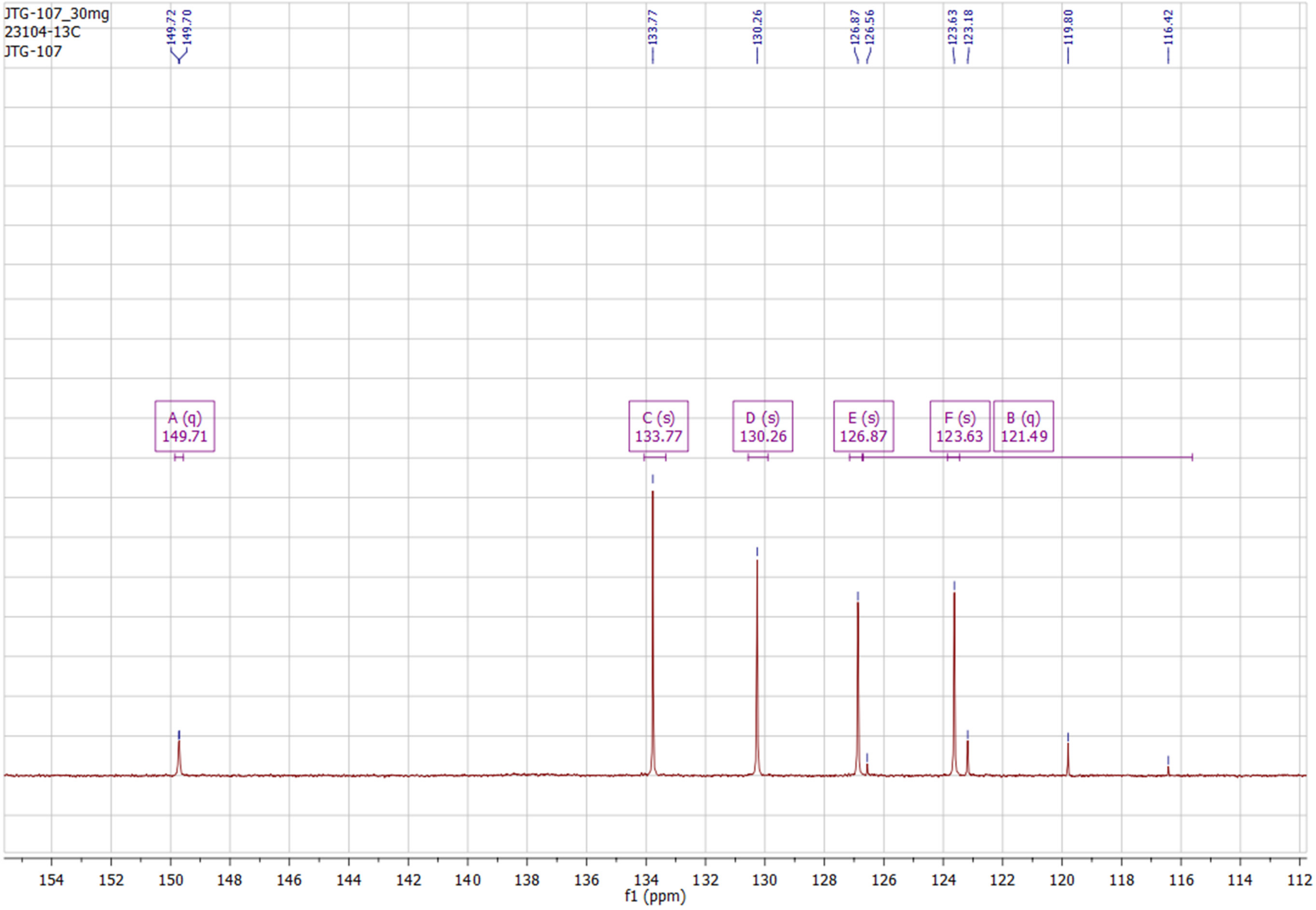

Figure S26. ^{13}C-NMR spectrum of **2** in $(CD_3)_2CO$ + a drop of D_2O (expansion).

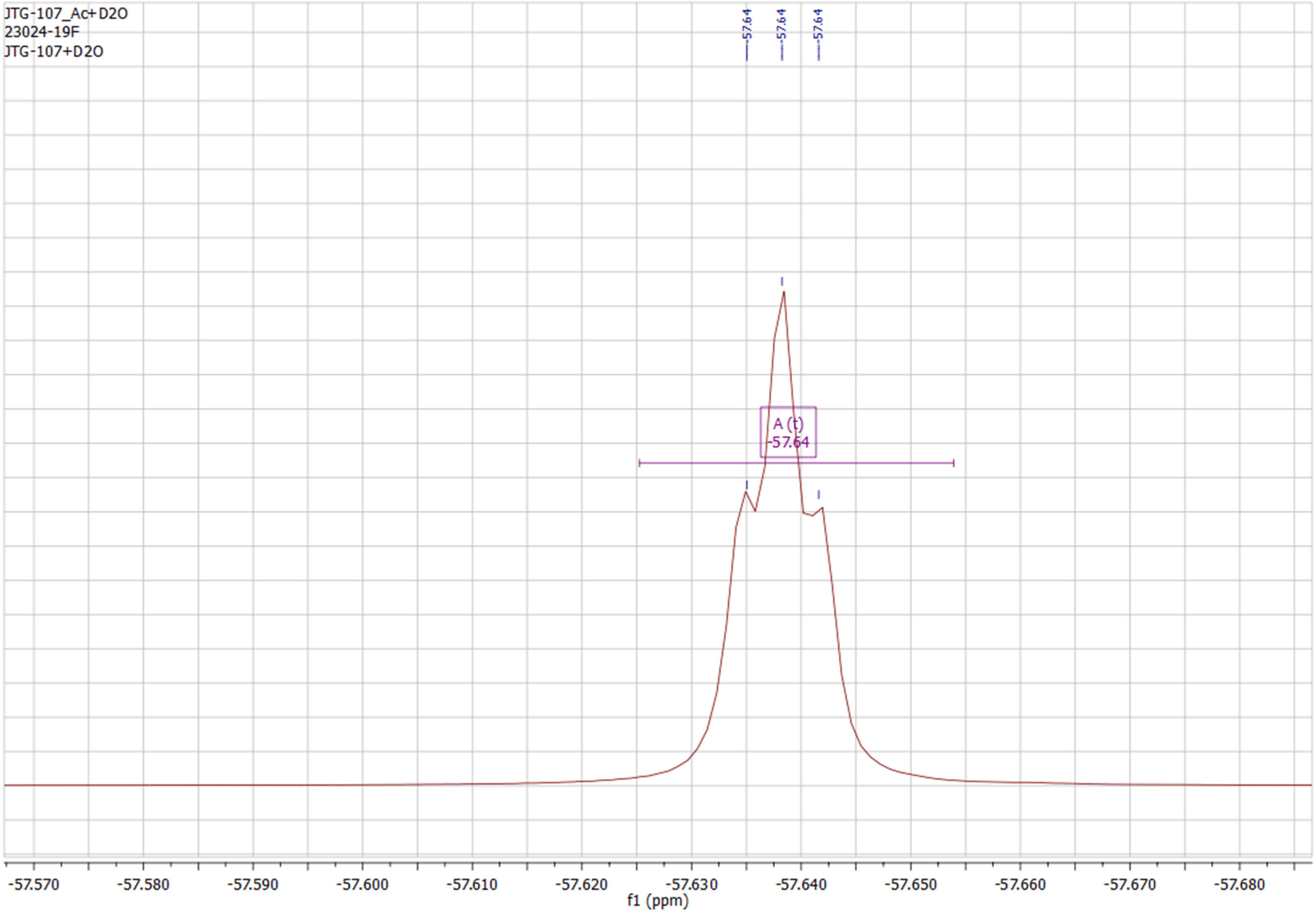

Figure S27. ^{19}F-NMR spectrum of **2** in $(CD_3)_2CO$ + a drop of D_2O (expansion).

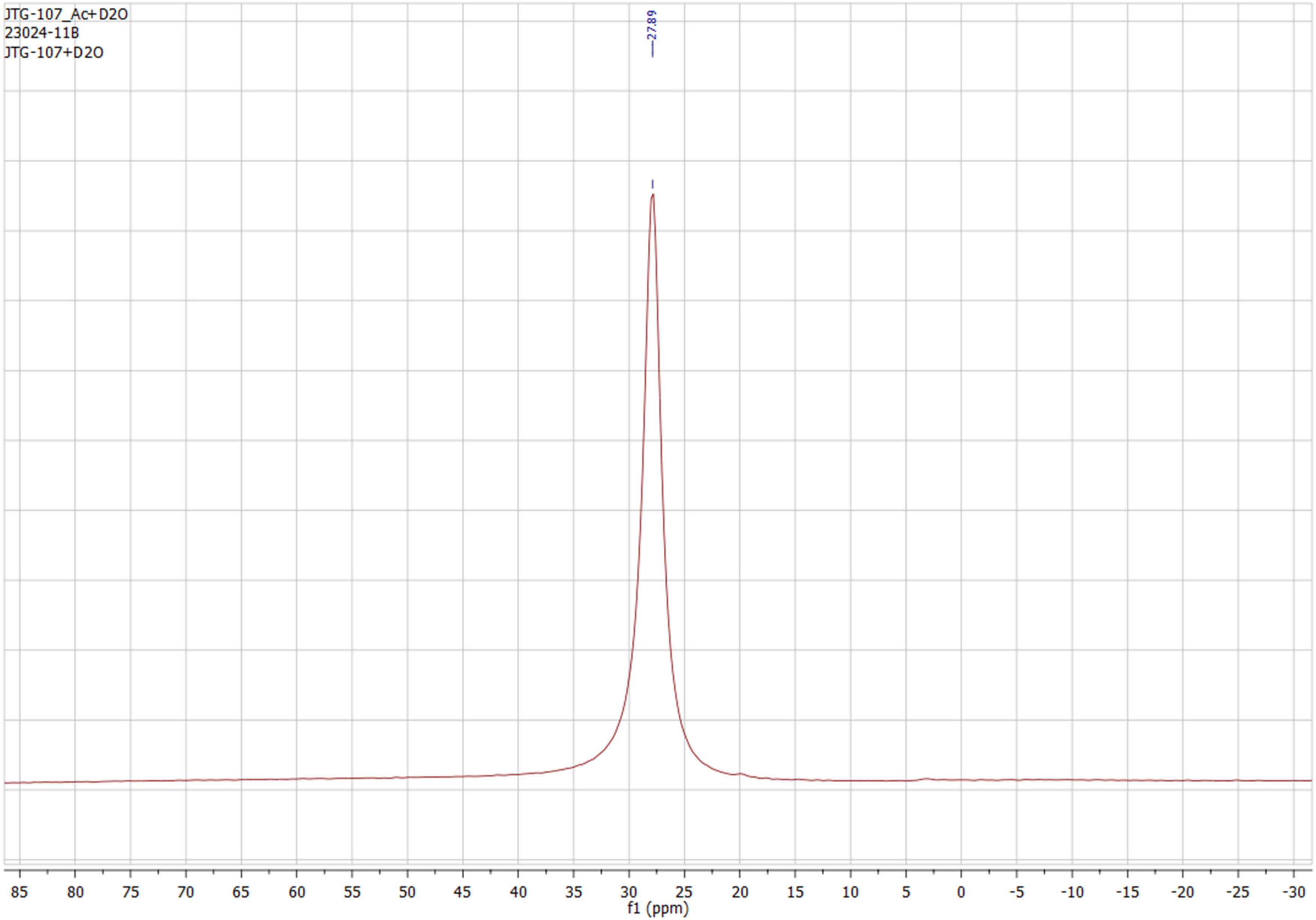

Figure S28. ^{19}F-NMR spectrum of **2** in $(CD_3)_2CO$ + a drop of D_2O (expansion).

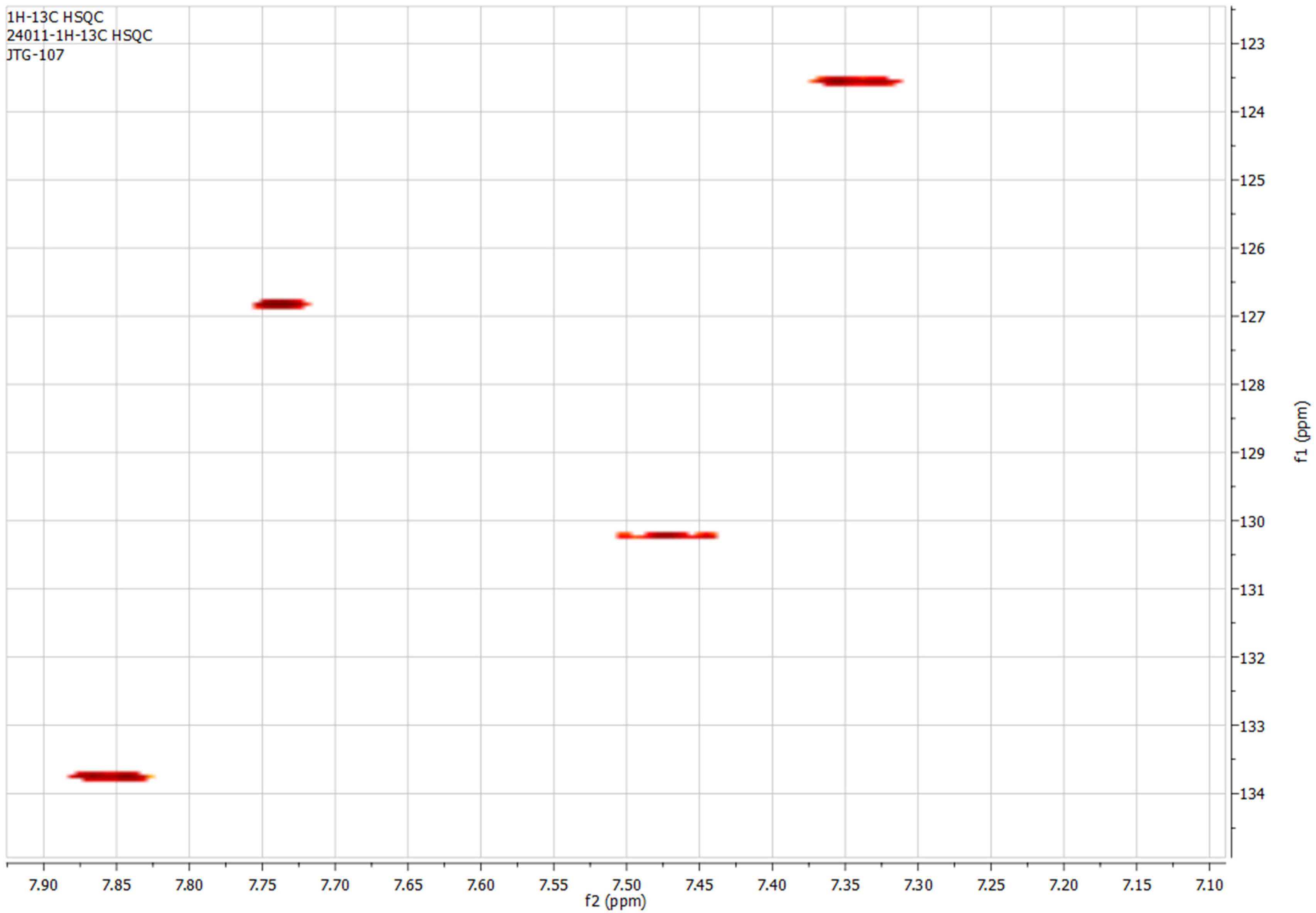

Figure S29. HSQC spectrum of **2** in $(CD_3)_2CO$ + a drop of D_2O.

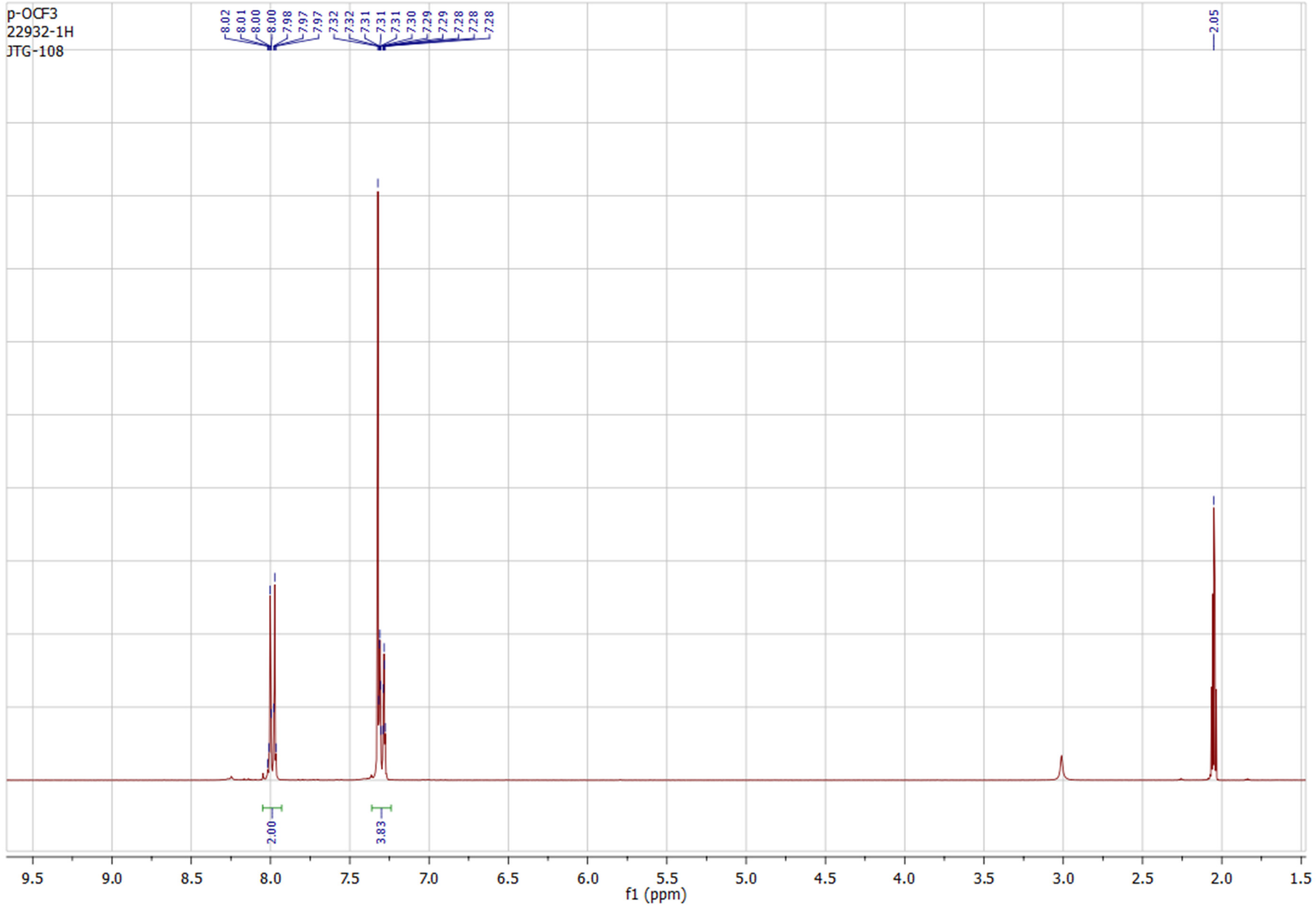

Figure S30. ^{1}H-NMR spectrum of **3** in $(CD_3)_2CO$ (full).

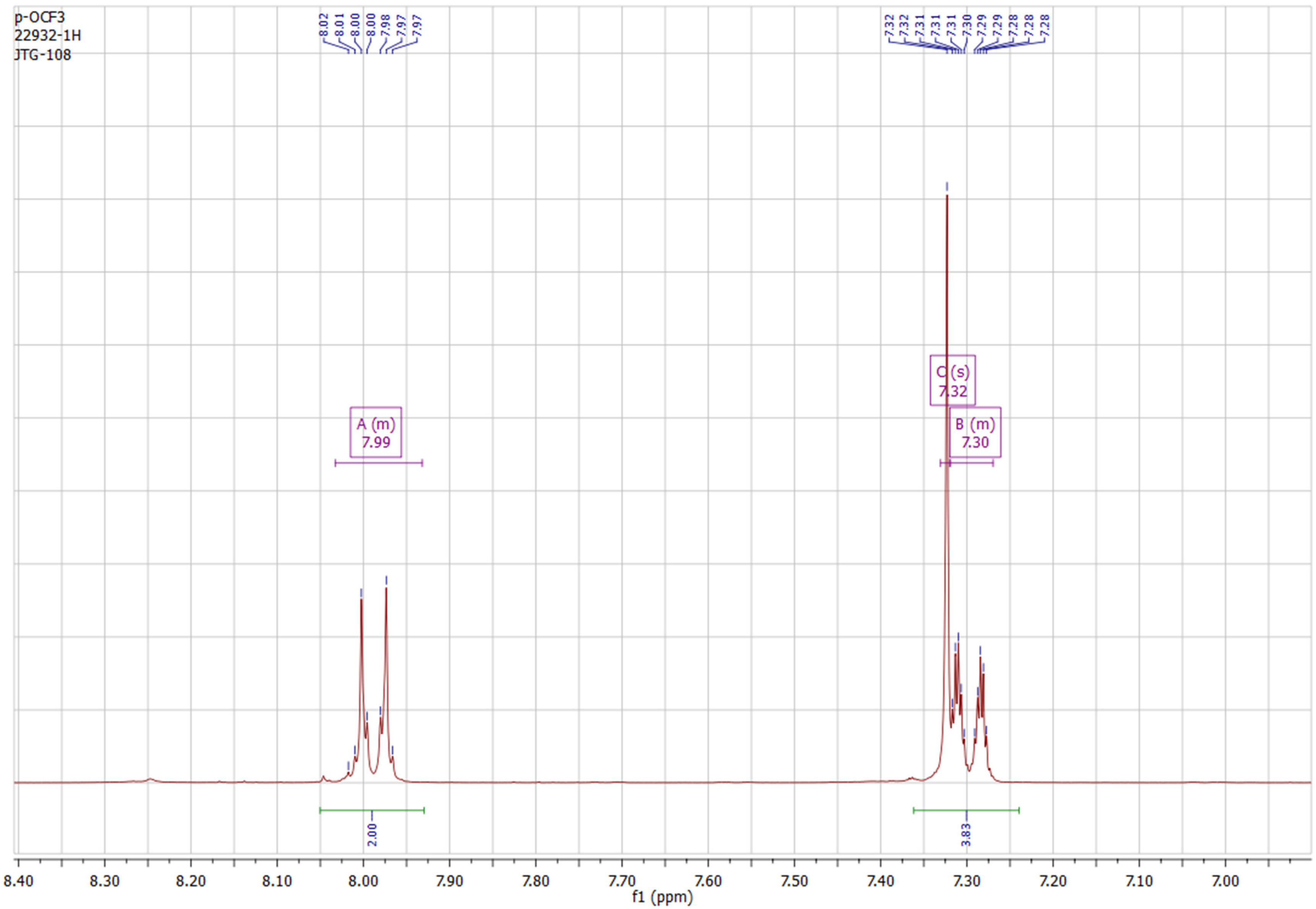

Figure S31. ^{1}H-NMR spectrum of **3** in $(CD_3)_2CO$ (expansion).

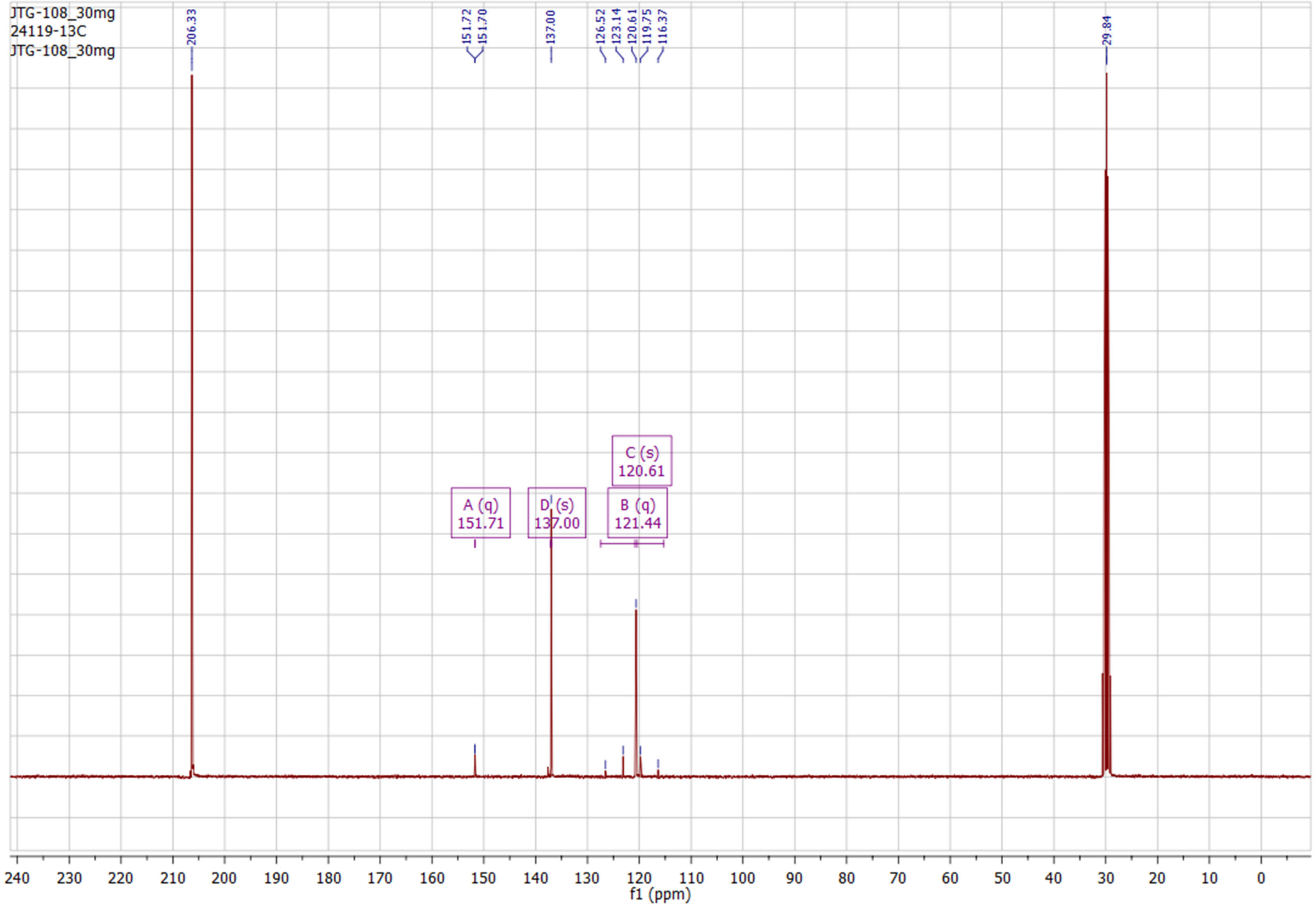

Figure S32. ^{13}C-NMR spectrum of **3** in $(CD_3)_2CO$ (full).

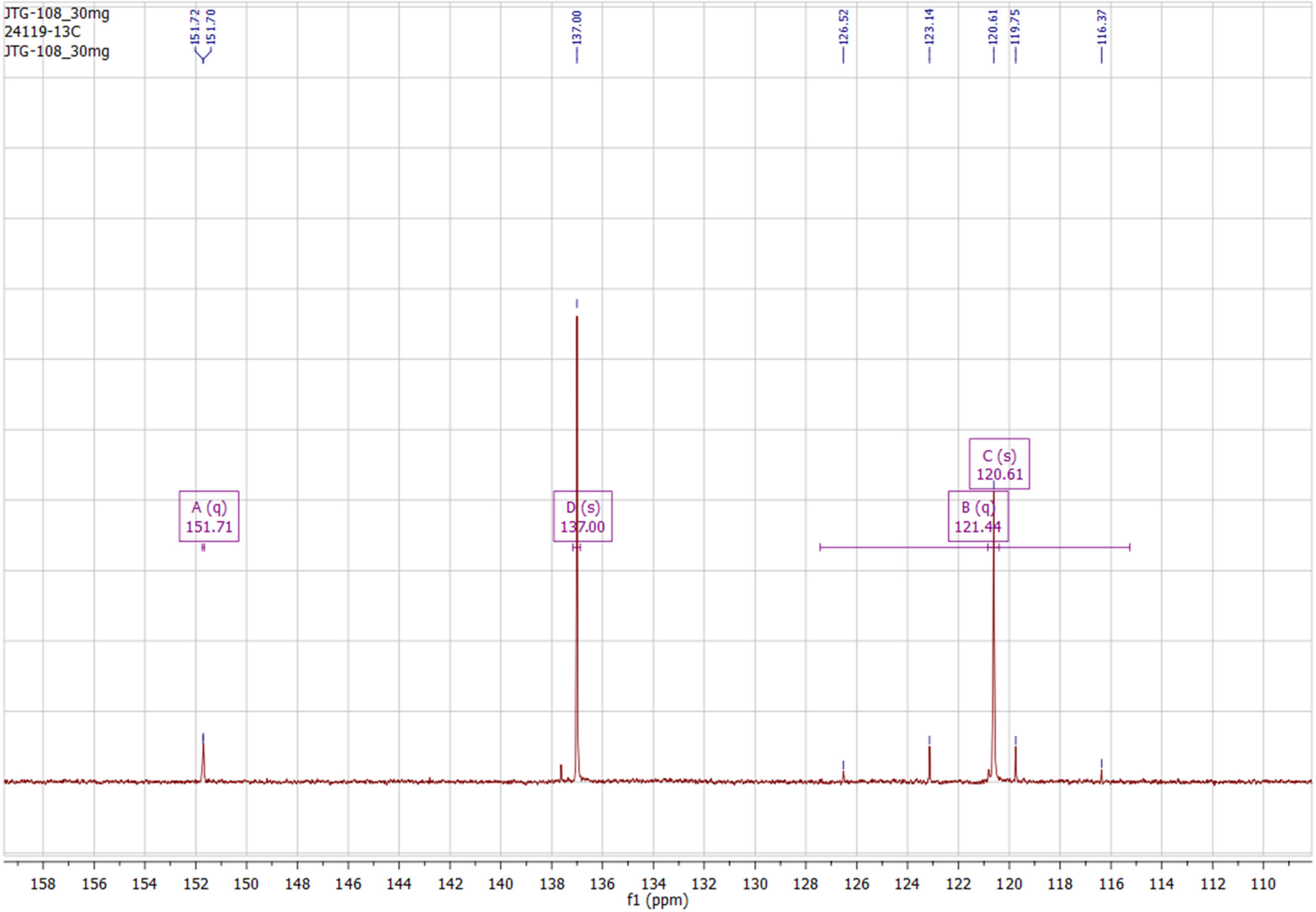

Figure S33. ^{1}H-NMR spectrum of **3** in $(CD_3)_2CO$ (expansion).

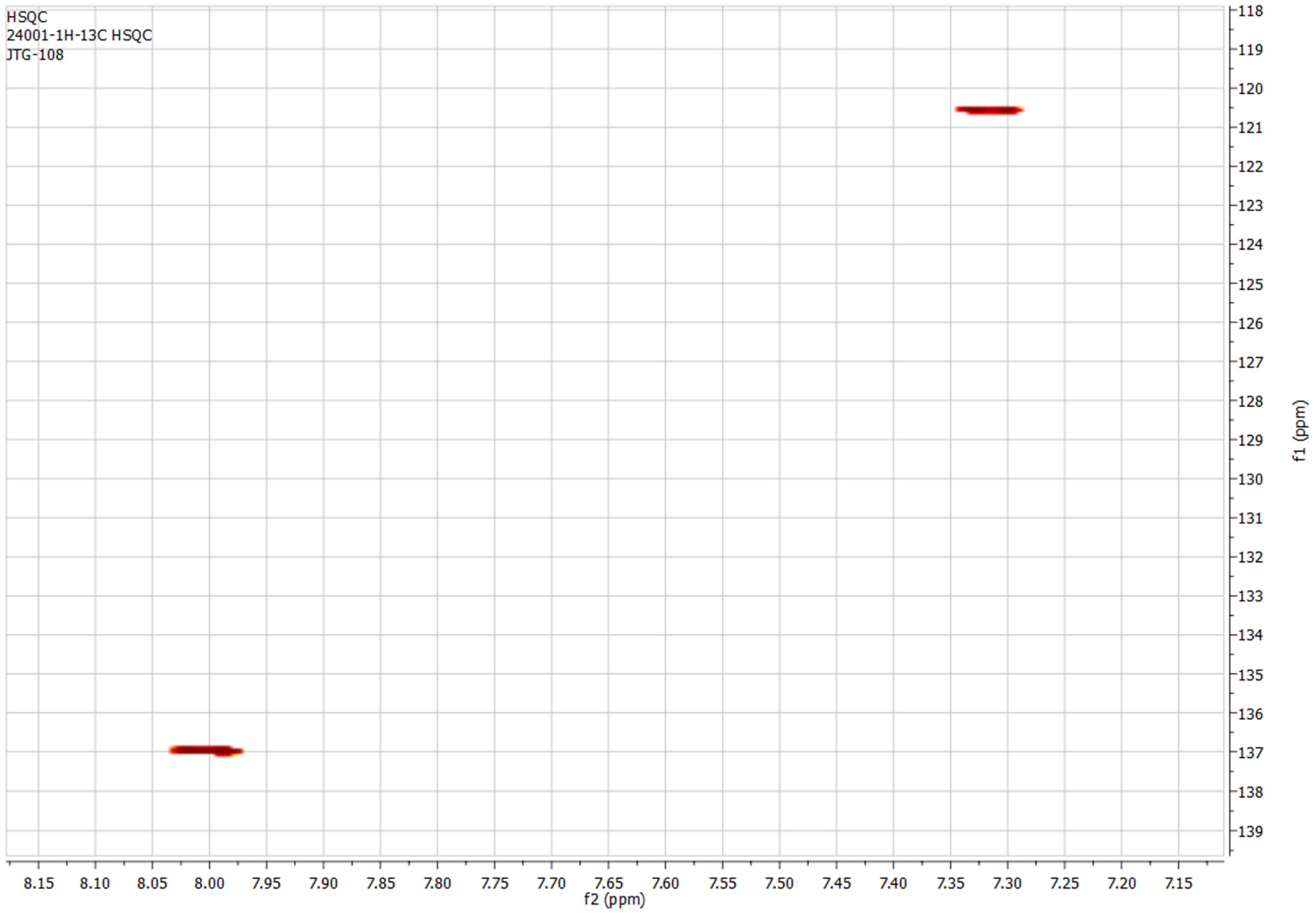

Figure S34. HSQC spectrum of **3** in $(CD_3)_2CO$.

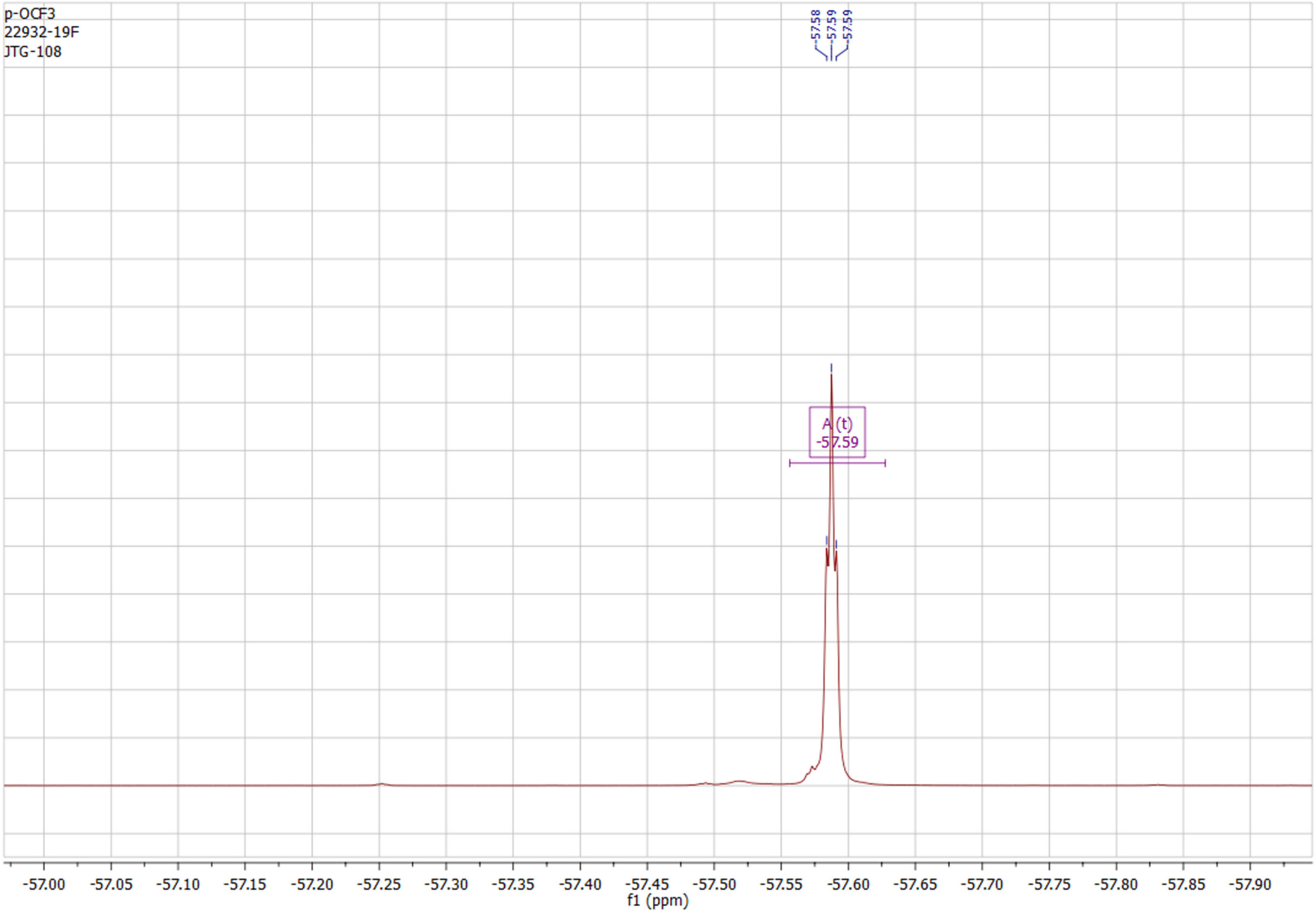

Figure S35. ^{19}F-NMR spectrum of **3** in $(CD_3)_2CO$.

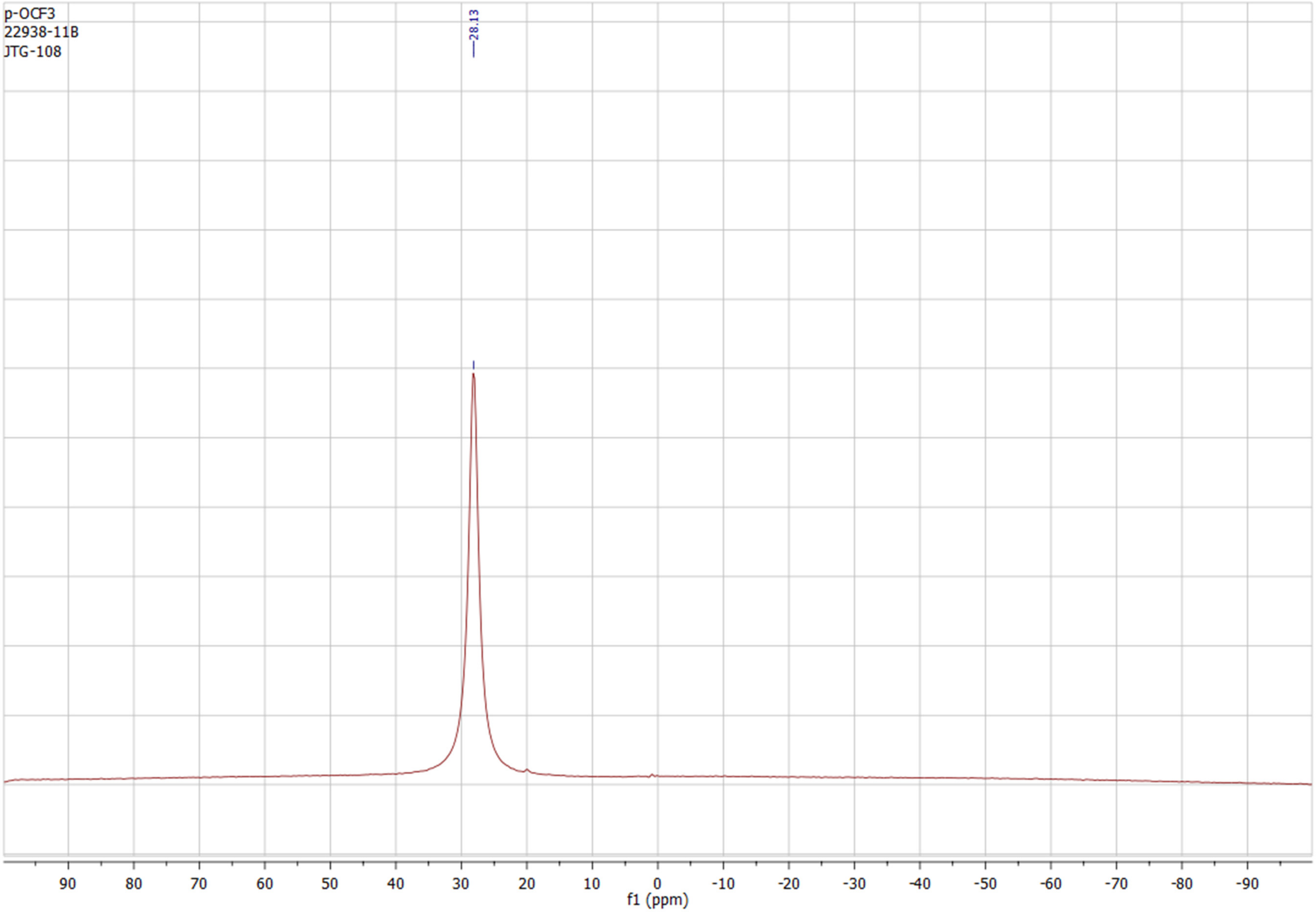

Figure S36. ^{11}B-NMR spectrum of **3** in $(CD_3)_2CO$.

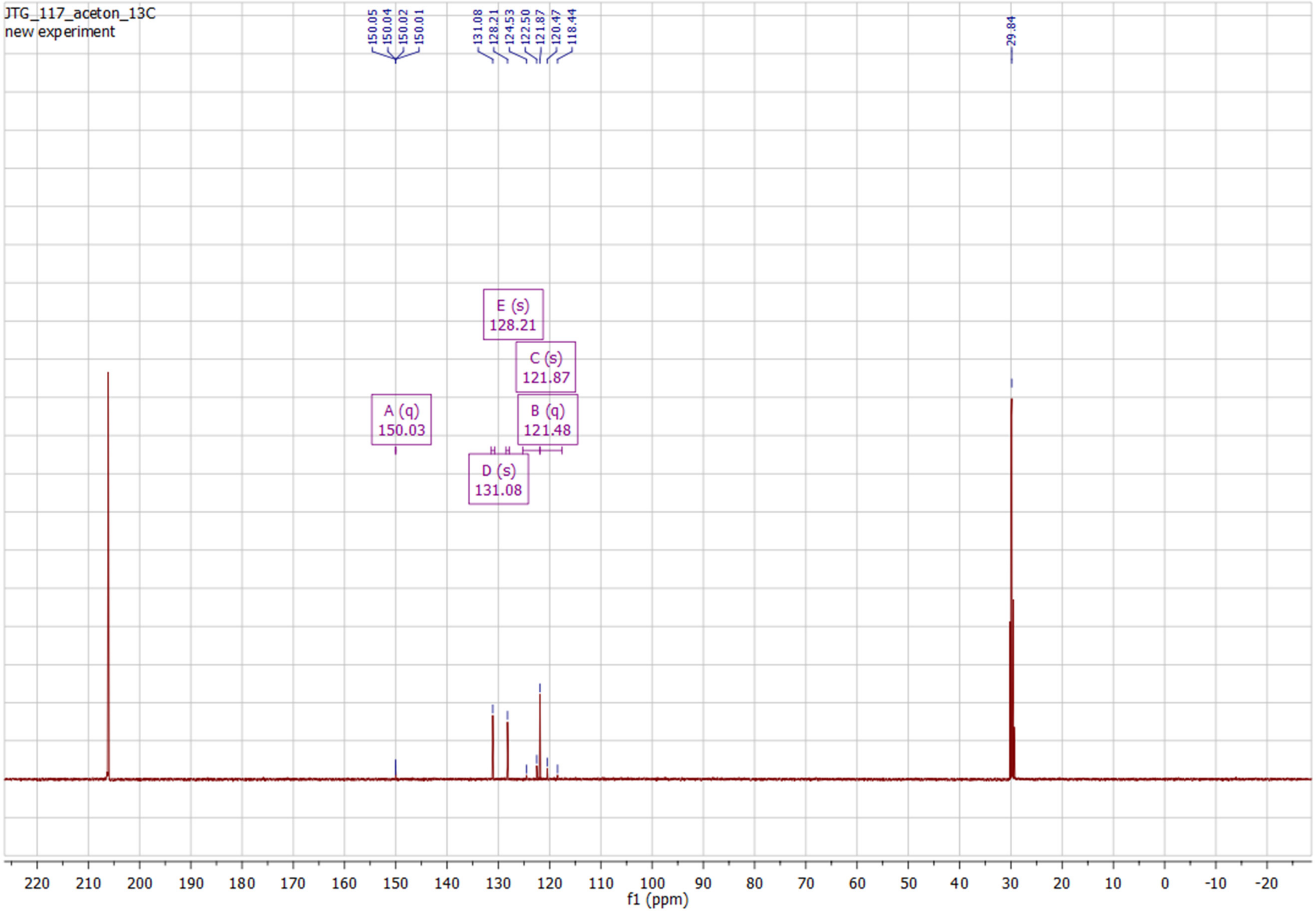

Figure S37. ^{13}C-NMR spectrum of ααα-trifluoroanisole in $(CD_3)_2CO$.
